# Bacterial genome-encoded ParMs

**DOI:** 10.1016/j.jbc.2025.110351

**Published:** 2025-06-09

**Authors:** Samson Ali, Adrian Koh, David Popp, Kotaro Tanaka, Yoshihito Kitaoku, Naoyuki Miyazaki, Kenji Iwasaki, Kaoru Mitsuoka, Robert C. Robinson, Akihiro Narita

**Affiliations:** 1 Research Institute for Interdisciplinary Science, Okayama University, Okayama, Japan; 2 Institute of Molecular and Cell Biology, A∗STAR (Agency for Science, Technology and Research), Biopolis, Singapore; 3 Graduate School for Integrative Sciences and Engineering, National University of Singapore, Singapore; 4 Cellular and Structural Physiology Institute (CeSPI), Nagoya University, Nagoya, Japan; 5 Division of Biological Science, Graduate School of Science, Nagoya University, Nagoya, Japan; 6 The Laboratory of Protein Synthesis and Expression, Institute for Protein Research, Osaka University, Suita, Japan; 7 Life Science Center for Survival Dynamics, Tsukuba Advanced Research Alliance (TARA), University of Tsukuba, Tsukuba, Japan; 8 Research Center for Ultra-High Voltage Electron Microscopy, Osaka University, Osaka, Japan; 9 School of Biomolecular Science and Engineering (BSE), Vidyasirimedhi Institute of Science and Technology (VISTEC), Rayong, Thailand

**Keywords:** DNA segregation, nucleotide hydrolysis, plasmid, ParM, ParCMR system

## Abstract

ParMs generally exist on low-copy number plasmids where they contribute to plasmid segregation and stable inheritance. We carried out bioinformatics analysis, which indicated that ParM genes are not only confined to plasmids but are also occasionally found on genomes. Here we report the discovery and characterization of two chromosome-encoded ParMs (cParMs) from the genomes of *Desulfitobacterium hafniense* (*Dh*-cParM1) and *Clostridium botulinum* (*Cb*-cParM). Both cParMs form filaments, exhibit nucleotide hydrolysis, and possess characteristic ParM subunit structures. *Dh*-cParM1 forms single and tightly coupled double filaments and is highly conserved on the chromosomes of five of six *Desulfitobacterium* species. Interestingly, these bacteria have not been reported to harbor plasmids. *Cb*-cParM possesses unique properties. Its filaments were stable after nucleotide hydrolysis and Pi release, and its ParR (*Cb*-cParR) did not affect the initial phase of *Cb*-cParM polymerization but displayed properties of a depolymerization factor for mature filaments. These results indicate functional, polymerizing ParMs can be encoded on genomes, suggesting that ParM roles may extend to other functions beyond plasmid segregation.

In contrast to the random diffusion mode of segregation for high copy number plasmids, low copy number plasmids require an active plasmid maintenance system to ensure equal distribution to daughter cells during cell division (1, 2). Polymerizing ParM proteins belong to the ParCMR system, one of the three known types of segregation or partitioning (par) systems that aid plasmid inheritance. ParM was originally discovered from the 100 kilobase pair multidrug resistant low-copy-number *E. coli* R1 plasmid (ParM-R1) (3, 4). Its polymerization provides the driving force to push two plasmids apart by binding to an adaptor protein, ParR which also binds to *parC*, a centromere-like DNA sequence located on the plasmid (5). Thus, ParM can partition the two plasmids to the two extremes of the cell leading to faithful inheritance (4, 6, 7, 8). The ParCMR systems are highly divergent, and a wide range of dynamics, nucleotide dependencies, and ParM filament architectures have been observed for the small number of systems studied to date (9, 10, 11, 12).

ParM homologs were initially thought to be restricted to plasmids of a close group of γ-proteobacteria due to high sequence variation and the limited number of ParMs characterized at the protein level. “ParM” genes from Firmicutes and cyanobacteria were postulated to represent other classes of bacterial actin-like proteins, rather than ParM orthologs (13). The recent characterization of a number of actin-like proteins or “ParMs” encoded on plasmids of Firmicutes bacteria such as, pSK41 from *Staphylococcus aureus*, BtParM from *Bacillus thuringiensis*, Alp7A and AlfA both from *Bacillus subtills*, and pCBH ParM filament from *Clostridium botulinum* have all indicated properties and features of ParMs similar to those of γ-proteobacteria (10, 11, 14, 15, 16, 17, 18). These properties include a closed beta-barrel domain, a distinguishing feature of ParMs amongst the actin superfamily; filament formation in a nucleotide-dependent manner; and ParR and *parC* sequences located near the ParM sequence on the plasmids (11), which led to the realization that ParMs are widespread and diverse in bacteria.

More than 65% of all sequenced bacterial genomes possess a chromosomally encoded partitioning (*par*) locus (19). This par system (ParABS) encodes chromosomal analogs of the active plasmid partitioning systems (ParCMR), which are involved in chromosome segregation (20). The ParABS system has been directly implicated in chromosome segregation in *Bacillus subtilis* and *Caulobacter crescentus* (21, 22). Unlike the “pushing” mechanism for plasmid segregation provided by polymerization of the actin-like ParM, the ParABS system operates through a binding and release mechanism along a concentration gradient. The ParABS system is composed of a deviant Walker-type fold NTPase (ParA) and a centromere-like DNA sequence (parS) and a parS binding protein (ParB) (23, 24). The host cell nucleoid is used to position ATP-bound ParA dimers. Interaction between the DNA-bound ParA dimers and the ParB/*parS* complex leads to ATP-hydrolysis, the dissociation of ParA dimers, and the release of the ParB/*parS* complex (24). A concentration gradient of DNA-bound ParA dimers, combined with the ATP-dependent binding and release, and flexibility in the DNA leads to the sequential passing of the ParB/*parS* section of the chromosome along DNA-bound ParA sites to the opposite cell pole to replication (25, 26).

However, ParABS systems are not present in many bacteria such as enteric bacteria, including *E. coli*, *Salmonella* species, *Haemophilus* species, and *Yersinia* species (19). Thus, it is possible that undiscovered active chromosome segregation systems exist. During our sequence searches for novel plasmid ParCMR systems, we noticed that several of these systems are encoded on chromosomes rather than on plasmids. Characterization at the molecular level of these chromosome-encoded ParCMR systems (cParCMR) has yet to be explored. Here we report the presence and characteristics of novel cParMs from *Desulfitobacterium species* and *C. botulinum*.

## Results

### Putative cParMR systems

**Figure 1:**
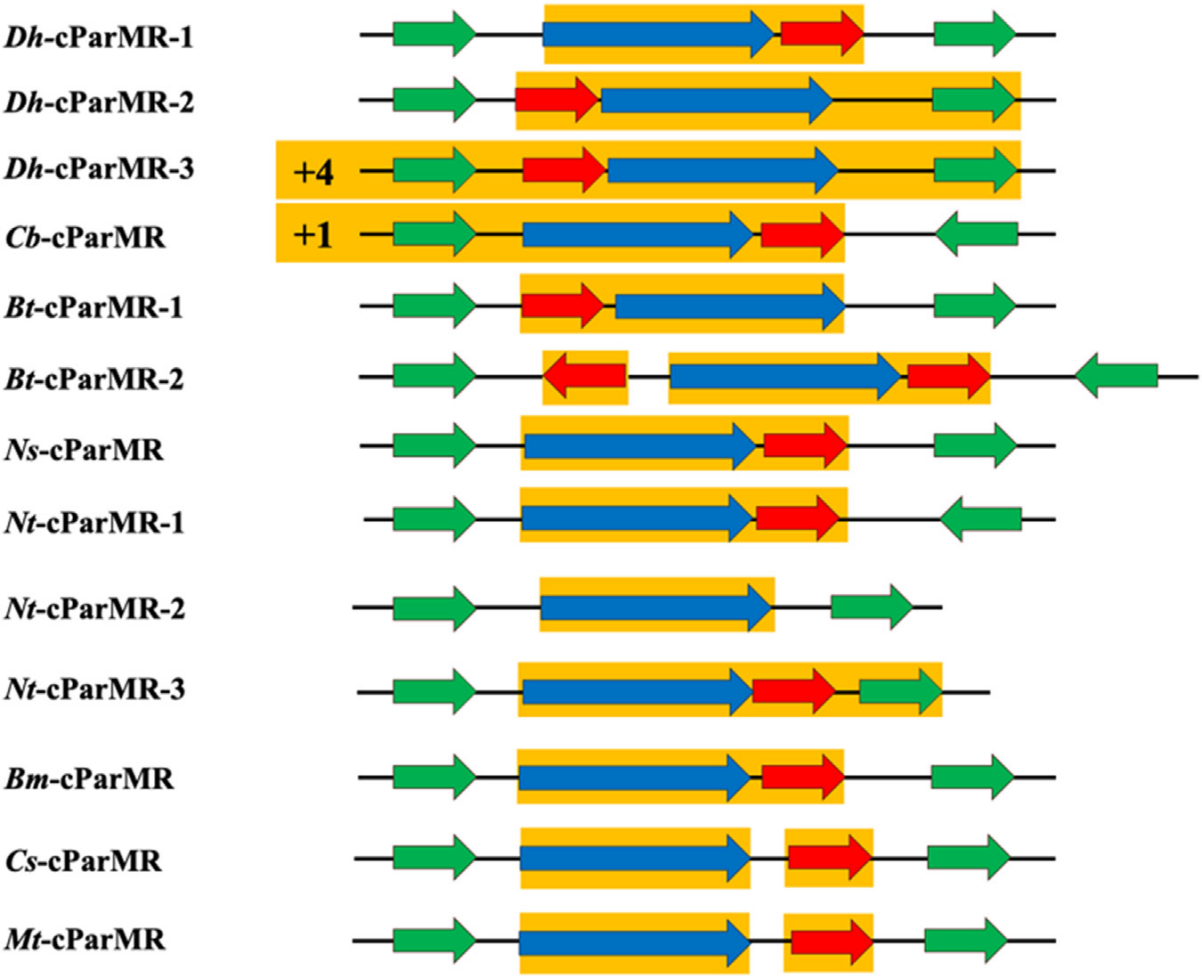
**Putative cParMR cassettes from different bacterial species.** Color coding: cParMs (*blue*), cParRs (*red*), and unrelated genes (*green*). Operons that contain cParMs or cParR are boxed in *orange*. The directions of the *arrows* show the direction of transcription. Three putative cParMR systems are encoded on the *Desulfitobacterium hafniense* Y51 chromosome (accession numbers: AP008230.1 or NC_007907.1, 5,727,534 bp): *Dh*-cParMR-1 (*Dh*-cParM1, WP_011460071.1; *Dh*-cParR1, WP_011460070.1); *Dh*-ParMR-2 (*Dh*-cParM2, WP_011461902.1; *Dh*-cParR2, WP_041272685.1); and *Dh*-cParMR-3 (*Dh*-cParM3, WP_011461997.1, *Dh*-cParR3, WP_011461998.1). *Natranaerobius thermophilus* strain JW/NM-WN-LF (accession number: NZ_CP144221.1, 3,137,840 bp) also encodes 3 cParMR systems - Nt-cParMR-1 (Nt-cParM1, WP_148206872.1; Nt-cParR1, WP_012449064.1), Nt-cParMR-2 (Nt-cParM2, WP_012448769.1) and Nt-cParMR-3 (Nt-cParM3, WP_012446843.1; Nt-cParR3, WP_012446842.1). This *Natranaerobius thermophilus* strain has two associated plasmids, pNTHE01 and pNTHE02 (accession numbers and sizes: NC_010715.1, 17,207 bp and NC_010724.1, 8689 bp, respectively). *Bacillus* tropicus strain FDAARGOS_920 (accession number: NZ_CP065739.1, 5,298,747 bp) encodes two putative cParMR systems *Bt*-cParMR-1 (*Bt*-cParM1, WP_001968526.1; *Bt*-cParR1, WP_129075283.1) and *Bt*-cParMR-2 (*Bt*-cParM2, WP_000025611.1; *Bt*-cParR2a, WP_001978111.1; *Bt*-cParR2b, WP_001257752.1). Other bacterial genomes have single cParMR cassettes, such as *Caldicellulosiruptor saccharolyticus* DSM 8903 (accession number: NC_009437.1, 2,970,275 bp): *Cs*-cParMR cassette (*Cs*-cParM, WP_011916932.1; *Cs*-cParR, WP_011916933.1), *Burkholderia multivorans* ATCC 17616 chromosome 3 (accession number: CP000870.1, 919,806 bp): *Bm*-cParMR (*Bm*-cParM, ABX19247.1; *Bm*-cParR, ABX19246.1) and *Moorella thermoacetica* strain 39073-HH (accession number: CP031054.1, 2,645,661 bp): *Mt*-cParMR cassette (*Mt*-cParM, WP_011391888.1; *Mt*-cParR, WP_053104303.1) and no associated plasmids (29, 76).

From Blast sequence searches (27), we identified many putative cParMR systems encoded on chromosomes (Fig. 1). These were located by searching for actin-fold signature sequences against fully sequenced genomes and inspecting the surrounding area for potential ParR-like sequences. The positions of cParRs varied, either located up- or downstream, relative to the cParM. We found systems that appear to lack cParR, such as the *Natranaerobius thermophilus* strain JW/NM-WN-LF *Nt*-cParM-2 cassette, and others contain more than one putative cParR, such as the *Bacillus tropicus* strain FDAARGOS_920 *Bt*-cParMR-2 cassette. Some chromosomes encode more than one potential cParMR system, as seen for *Desulfitobacterium hafniense* strain Y51 *Dh*-cParMR-1,2,3 cassettes, *N. thermophilus* strain JW/NM-WN-LF 1 *Nt*-cParMR-1,2,3 cassettes, and *B. tropicus* strain FDAARGOS_920 *Bt*-cParMR-1,2 cassettes. *Nt*-cParM was not observed to be conserved on chromosomes from other *Natranaerobius* species; however, *Bt*-cParM and *Mt*-cParM (from *Moorella thermoacetica* strain 39073-HH) are conserved on several *Bacillus* and *Moorella* species chromosomes, respectively (Tables S1–S3). Some bacterial strains encoding cParMs (Fig. 1), such as *Desulfitobacterium* and *Moorella* species, do not harbor plasmids (28, 29) whereas plasmids are known for other species, such as *N. thermophilus* strain JW/NM-WN-LF. *Cb*-cParMR was located on the whole genome shotgun sequences or complete chromosomes of *C. botulinum* with genome sizes ranging from 3.9 to 4.3 mb. We confirmed the ParM fold of the cParMs by calculating the AlphaFold2 (30) predicted structures and inspecting them for the closed beta-barrel domain architecture (Fig. S1). We proceeded to characterize two cParMR systems, *Dh*-cParMR-1 and *Cb*-cParMR, to ascertain whether they have similar properties to plasmid-encoded systems.

### *Dh*-cParMR-1 cassette and sequence conservation in *Desulfitobacterium* species

ParM from the *D. hafniense* Y51 bacterium *Dh*-cParMR-1 cassette is annotated as a potential chromosome segregating cParM in the NCBI sequence database, despite the gene residing on the 5,727,534-bp circular chromosome (28). The cParM protein sequence is highly conserved among five of the six *Desulfitobacterium* species - *metallireducens*, *dichloroeliminans*, *dehalogenans*, *chlororespirans,* and *hafniense* (Table 1), and syntenically conserved cParMR clusters are found in *Desulfitobacterium* genomes (Fig. S2). Additional copy or copies of ParMs were identified in limited genomes. In total, three copies for *D. hafniense*, two copies for *Desulfitobacterium dichloroeliminans* and *Desulfitobacterium chlororespirans*, and only single copy for the remaining two species were identified. cParM1 sequences are highly conserved throughout the *Desulfitobacterium* genomes (Fig. S3). Conserved cParR genes are found downstream of cParM1 genes. cParRs also accompany the additional copies of cParMs. However, the sequence similarities between the canonical cParRs and the cParRs from different loci are limited (data not shown). Despite this, AlphaFold3 (31) predicted canonical ribbon-helix-helix motifs within these sequences, which is a typical DNA-binding motif that is also adopted by ParRs (32). According to the surrounding genetic context and the conservation of their predicted folds, we annotated these distant relatives as potential cParRs.

**Table 1:** *Dh*-cParM1 is highly conserved among the *Desulfitobacterium* species

Sequence identity	Hafniense	Chlororespirans	Dichloroeliminans	Dehalogenans	Metallireducens
hafniense	100.00	98.4	94.6	92.4	84.6
chlororespirans	98.4	100.0	92.7	94.9	84.3
dichloroeliminans	94.6	92.7	100.0	93.2	85.1
dehalogenans	92.4	94.9	93.2	100.0	84.8
metallireducens	84.6	84.3	85.1	84.8	100.0

Sequence identities as percentages among five out of the six *Desulfitobacterium species.* cParM was not found by sequence homology searches in *Desulfitobacterium aromaticivoran* genomes.

When *Desulfitobacterium* individual cParM-coding sequences (CDSs) were compared, pairs of cParM CDSs showed between 84.2 to 98.3% identity. These ranked as the 79.7 to 96.4% top percentiles compared to all homologous CDSs in pairs of genomes (Fig. S4). The lowest percentile (79.7%) was observed when *D. hafniense* cParM CDS were aligned against *D. chlororespirans* CDSs, which has the highest sequence identity (98.3%) for their cParMs. Since these two species are the most closely related among the species compared (33), the relatively low percentile is due to the largest bin comprised of mostly identical sequences (Fig. S3). Accordingly, among the conserved CDSs in *Desulfitobacterium* bacteria genomes, we suggest that cParM1 sequences are one of the most highly conserved sequences. This high sequence conservation implies an indispensable function in these bacteria. These homologs are variously annotated as “chromosome segregation protein ParM” or “plasmid segregation actin-type ATPase ParM” in NCBI databases. We attempted the *E. coli* expression followed by purification of the *Dh*-cParM1 and *Dh*-cParR1, however only *Dh*-cParM1 produced sufficient protein for characterization.

### *Dh*-cParM1 filaments show polymorphism in architecture

**Figure 2:**
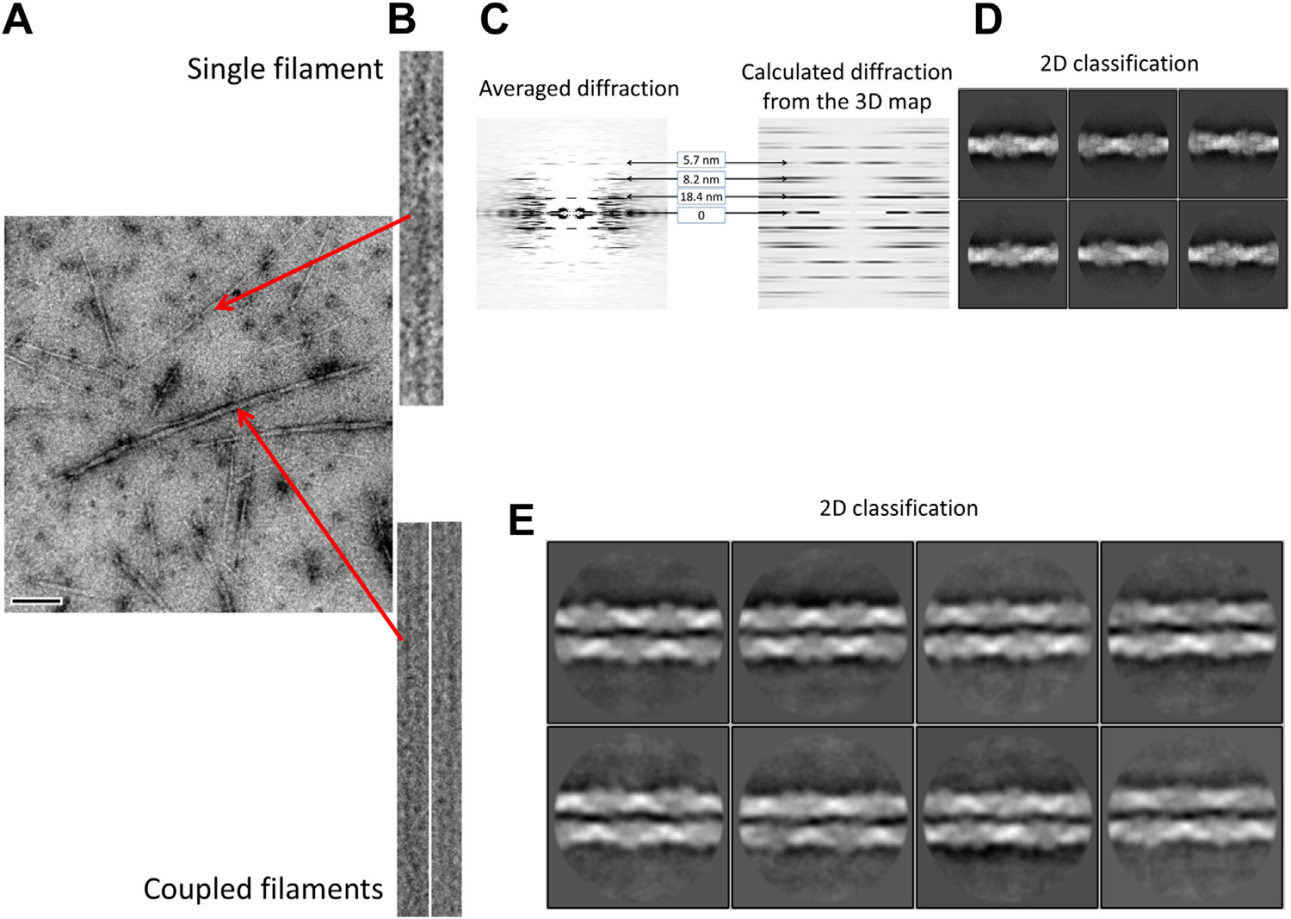
**Variations in helical parameters of the two types of *Dh*-cParM1 filaments.***A*, electron micrograph of a negatively stained sample displaying the two types of *Dh*-cParM1 filament morphologies – single and coupled filaments. *B*, extraction of two distinct filament types from the same micrograph. The single and coupled filaments were extracted and straightened for helical parameter determinations. *Top*: single filament and *Lower*: coupled filament. *C*, the averaged diffraction pattern of single filament morphology from negatively stained micrographs (*left image*) and from reconstructed 3D structure (*right image*). *D*, 2D classification of *Dh*-cParM1 extracted particles of single filaments from cryoEM micrographs. *E*, 2D class averages of the coupled filament morphology from cryoEM micrographs. Detailed analysis showed these structures comprise two single filaments lying side by side, which sometimes taper at the ends (Fig. S6).

**Figure 3:**
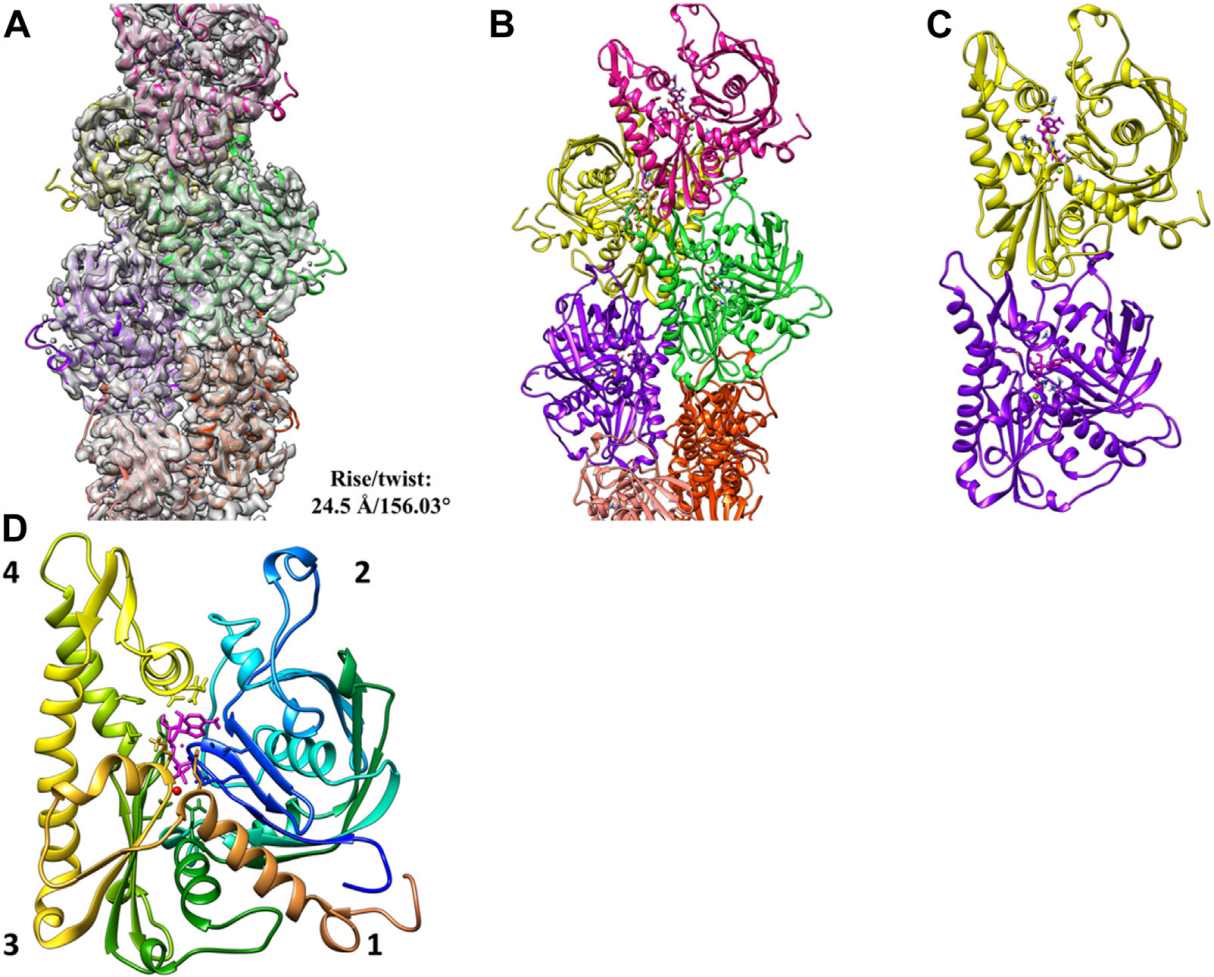
***Dh*-cParM1 shows a double-helical single filament structure.***A*, protomers fit into the 4 Å resolution density map of *Dh*-cParM1 single filament. *B*, a short segment of the *Dh*-cParM1 filament model. Protomer structures are shown with different colors in the filament. *C*, a dimer of *Dh*-cParM1 protomer showing the longitudinal contacts *D*, *Dh*-cParM1 monomer displaying its four subdomains (numbered) similar to actin and other ParMs. The nucleotide which is likely to be ADP from hydrolysis of ATP is indicated in magenta and the magnesium ion in *red*.

*Dh*-cParM1 polymerization induced by ATP was followed by light scattering (Fig. S5*A*). The increase in light scattering was confirmed to be due to filament formation, which was imaged by electron microscopy of negatively stained samples. Filaments were induced by the addition of ATP, GTP, and non-hydrolysable nucleotides (Fig. S6, *A*–*D*) (33). Sedimentation studies indicated that ADP and GDP have a lower ability to induce polymerization relative to ATP and GTP, respectively, with no discernable increase in sedimented *Dh*-cParM1 on the addition of GDP (Fig. S5*B*). The estimated residual concentration of *Dh*-cParM1 in the supernatants was approximately 4 to 5 μM for both ATP and GTP (Fig. S5*B*), comparable to the critical concentration of ParM-R1 (34). On the same EM micrograph and under the same buffer conditions, two filament morphologies were observed: single filaments and tightly coupled double filaments (Fig. 2*A*). Fourier transform analysis of the *Dh*-cParM1 filaments in the micrographs indicated helical parameters of twist/rise of 156.0°/23.5 Å for the single filament (Fig. 2, *B* and *C*). Since this morphology was more abundant, using cryo-EM we were able to achieve a high-resolution structure reconstruction. Analysis of the 2D classes showed clear secondary structure features highlighting the variations in the filament morphologies (Fig. 2*D*). The single filaments displayed structural features resembling that observed for eukaryotic F-actin and ParM-R1. From a total of 2845 images and a final selection of 71,060 particles, a 4.0 Å resolution density map of a *Dh*-cParM1 single filament constructed from two parallel strands was reconstructed displaying a helical rise and twist of 24.5 Å and 156.03°, respectively (Fig. 3, *A* and *B*). Its short helical repeat is about half that of F-actin or ParM-R1 resulting in a more twisted structure. The two protofilaments are more tightly packed than those of ParM-R1 and pCBH ParMs (9, 11, 18).

We suspected that the tightly coupled *Dh*-cParM1 double filaments may be produced from the side-by-side interaction between two single filaments (Figs. 2, *A* and *E*, and S6). The distances between the two filaments were not uniform throughout the structure, often observed to be tapering at the ends. 2D class averaging in RELION 4.0 (with T = 0.5) (35) confirmed the coupled filament structures (Fig. 2*E*). ParM-R1 can be induced by crowding agents to form similar structures, known as doublets, which show no supercoiling or twisting (9). The interactions between the two *Dh*-cParM1 filaments of this morphology are very similar to those of ParM-R1 doublets. They both showed no supercoiling or twisting of the double filaments. *Dh*-cParM1 spontaneously forms these two filament morphologies without the need for crowding agents. *Dh*-cParM1’s ability to form polymorphs of this nature is a unique feature among ParMs, which may have implications for its function.

### *Dh*-cParM1 filament structure

We employed AlphaFold2 models as starting models to interpret the cryoEM density maps. Refining these models against the cryoEM density maps, protomer structures characterized by good statistics were generated (Fig. 3, *A*–*D* and Table S4). As observed for other ParM protomers, the analogous subdomain 2 region to the DNase I binding loop in actin (36, 37, 38) was clearly observed in the density as well as the characteristic ParM closed beta-barrel in subdomain 1 (Fig. 3*D*). Due to the limited resolution, we cannot unambiguously determine the state of the nucleotide bound in this structure. However, ADP appears to fit better into the cryoEM density map than ATP, which is consistent with polymerization initiating hydrolysis of ATP (Fig. S7*A*). The interacting residues surrounding the nucleotide bear similarity to those of ParM-R1 (Fig. S8). The hydrophobic residues of subdomain 2 interact with the hydrophobic pocket of subdomains 1 and 3 of the upper subunits in a ball and socket manner, similar to that observed in p*CB*H ParM (11) (Fig. 3*C*). The intra-strand arrangements are mainly formed through lateral contacts, whereas the interstrand arrangements are held together by lateral contacts as observed in other ParMs (Fig. 3*B*). The single filament structure is constructed from two parallel, staggered protofilaments.

### *Cb*-cParCMR

**Figure 4:**
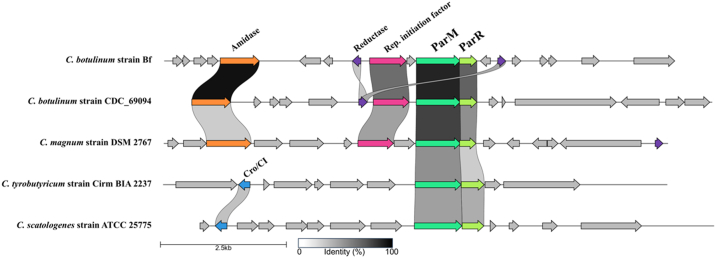
**Gene clusters of *Clostridium* species containing cParMR system.** Genes within 5000 bp of cParM are depicted using clinker & cluster map (55). *Clostridium* species containing homologous cParM sequences (identity cutoff = 50%) are aligned. Genes are depicted as *arrows*. Conserved genes, cParM (*light green*), cParR (*lime green*), putative replication initiation factor (*magenta*), sporulation-specific N-acetylmuramoyl-L-alanine amidase (*orange*), dihydrodipicolinate reductase (*purple*), and Cro/CI family transcriptional regulator-like protein (*blue*), are colored. Annotations are from GenBank records. c*parC* is not displayed, since a conserved c*parC* sequence was not identified among strains.

**Figure 5:**
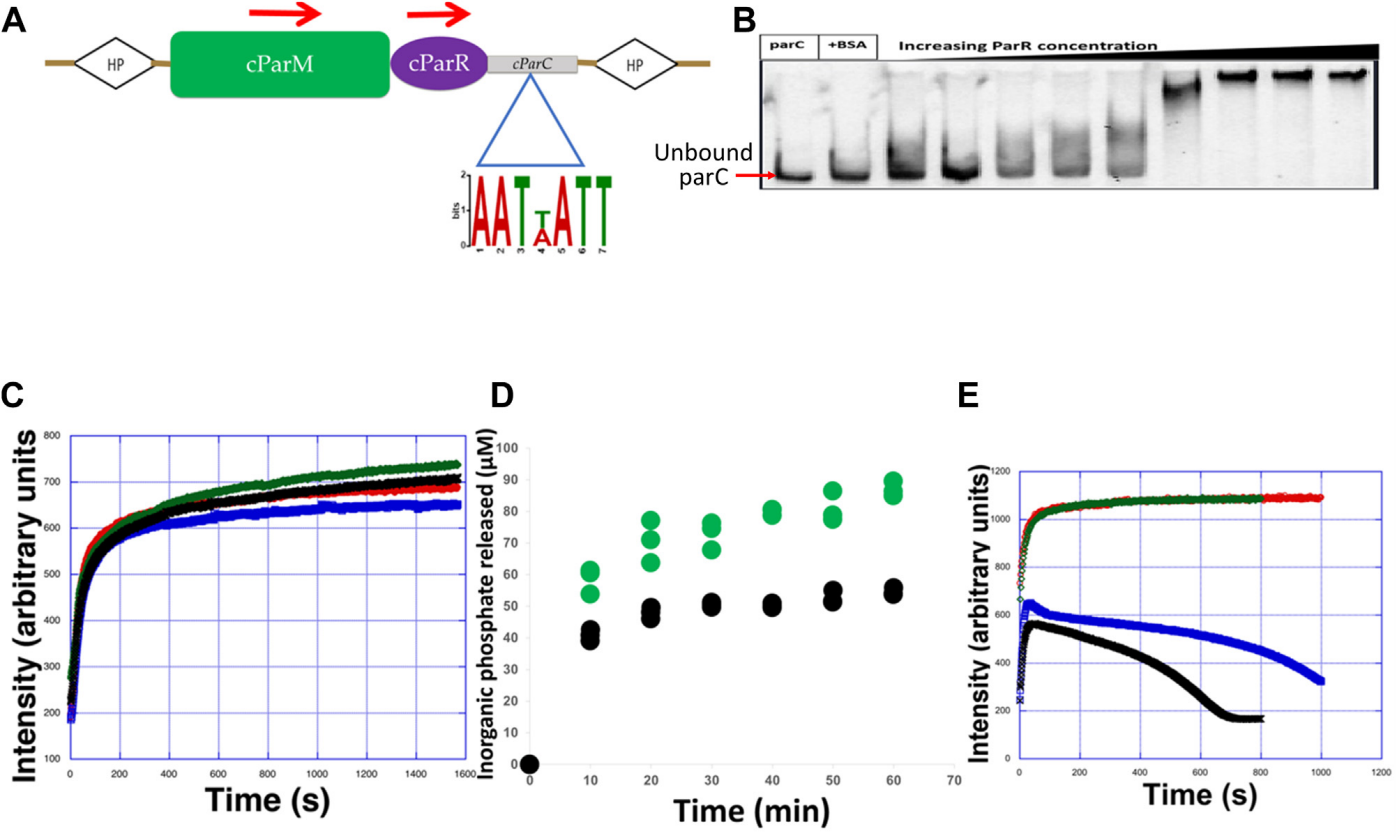
**Characterization of the *Cb*-cParCMR system.***A*, cParCMR system present on the genome of *C. botulinum* strain Bf. *Cb*-*cParC* includes three palindromic repeats. The *red arrows* above cParM and cParR indicate the direction of the transcription based on the operon analysis (Fig. 1). c*parC* is positioned after cParR sequence. HP represents hypothetical proteins on both sides of the cParCMR operon. *B*, electrophoretic mobility shift assay of *Cb*-*cParC* with 10 × to 1000 × molar excess of cParR. *C*, light scattering time courses of *Cb*-cParM polymerization. 10 μM *Cb*-cParM *green*, ATP; *black*, GTP; *red*, ADP; *blue*, GDP. *D*, Pi release from 10 μM *Cb*-cParM, *green* with ATP and *black* with GTP. Three measurements for each nucleotide are superimposed. *E*, light scattering time courses of *Cb*-cParM polymerization in the presence of *Cb*-cParR, *blue*, with ATP; *black*, with GTP; Corresponding time courses without *Cb*-cParR, *red*, with ATP; *green*, with GTP.

*Cb*-cParM (EDT87363.1) and *Cb*-cParR (EDT87283.1) sequences were identified through homology searches using BLAST on a whole-genome shotgun sequence of *C. botulinum* Bf (ABDP01000001.1) (Figs. 1 and 4). A putative *cparC* sequence containing palindromic sequence repeats was also found immediately upstream of *Cb*-cParMR (Fig. 5*A*). This *Cb*-c*parC* sequence is bound to *Cb*-cParR in an electrophoretic mobility shift assay (Fig. 5*B*), indicating that it acts as the *Cb*-cPar*C*. Gene clusters with high similarity to the sequence around *Cb*-cParCMR were found in the genomes of some other *Clostridium* strains and were not found on plasmids (Figs. 4 and S9). Therefore, at the current level of genome sequencing, we conclude that *Cb*-cParCMR is encoded on chromosomes from a limited set of *Clostridium* strains.

### Polymerization assay and cParR–cParM interactions

*Cb*-cParM polymerized with GTP, ATP, GDP, and ADP at similar rates as judged by an increase in light scattering over time (Fig. 5*C*). This is in stark contrast to *Dh*-cParM1 for which polymerization in the presence of ADP and GDP was largely reduced compared to ATP and GTP (Fig. S5*B*). After polymerization, *Cb*-cParM remained as a filament without undergoing bulk depolymerization judged by constant light scattering intensity (Fig. 5*C*), unlike previously studied ParMs (10, 11) and *Dh*-cParM1 which depolymerized slowly after polymerization (Fig. S5*A*). Supernatant *Cb*-cParM concentrations in a sedimentation assay indicated that the critical concentrations for polymerization were similar in the presence of the different nucleotides, 0.31 ± 0.02 μM, 1.81 ± 0.11 μM, 0.61 ± 0.01 μM, and 1.92 ± 0.06 μM with ATP, ADP, GTP, and GDP, respectively (Table 2, Figs. S10, and S11). The critical concentration dependence of *Cb*-cParM on the nucleotide state is considerably smaller than that of actin (39) or *E. coli* ParM-R1 (34). Continuous phosphate release beyond the concentration of the protein (10 μM *Cb*-cParM) was observed (Fig. 5*D*), suggesting subunit exchange from the ends of the filament after the initial polymerization, consistent with a process such as treadmilling (40, 41, 42).

**Table 2:** *Cb*-cParM critical concentrations calculated from pelleting assay described in Fig. S10

Conditions	Critical concentration (μM)
*Cb*-cParM + ATP	0.31 ± 0.02
*Cb*-cParM + ADP	1.81 ± 0.11
*Cb*-cParM + GTP	0.61 ± 0.01
*Cb*-cParM + GDP	1.92 ± 0.06

In the presence of *Cb*-cParR, initial polymerization of *Cb*-cParM proceeded normally (Fig. 5*E*) (43, 44). However, after the initial polymerization phase, *Cb*-cParR destabilized the *Cb*-cParM filaments and depolymerization occurred, indicating that *Cb*-cParR acts as a depolymerization factor (Fig. 5*E*). A sedimentation assay also indicated the destabilization of the *Cb*-cParM filament by *Cb*-cParR after 30 min incubation, which was not dependent on *Cb*-*parC* (Fig. S12).

### CryoEM imaging

**Figure 6:**
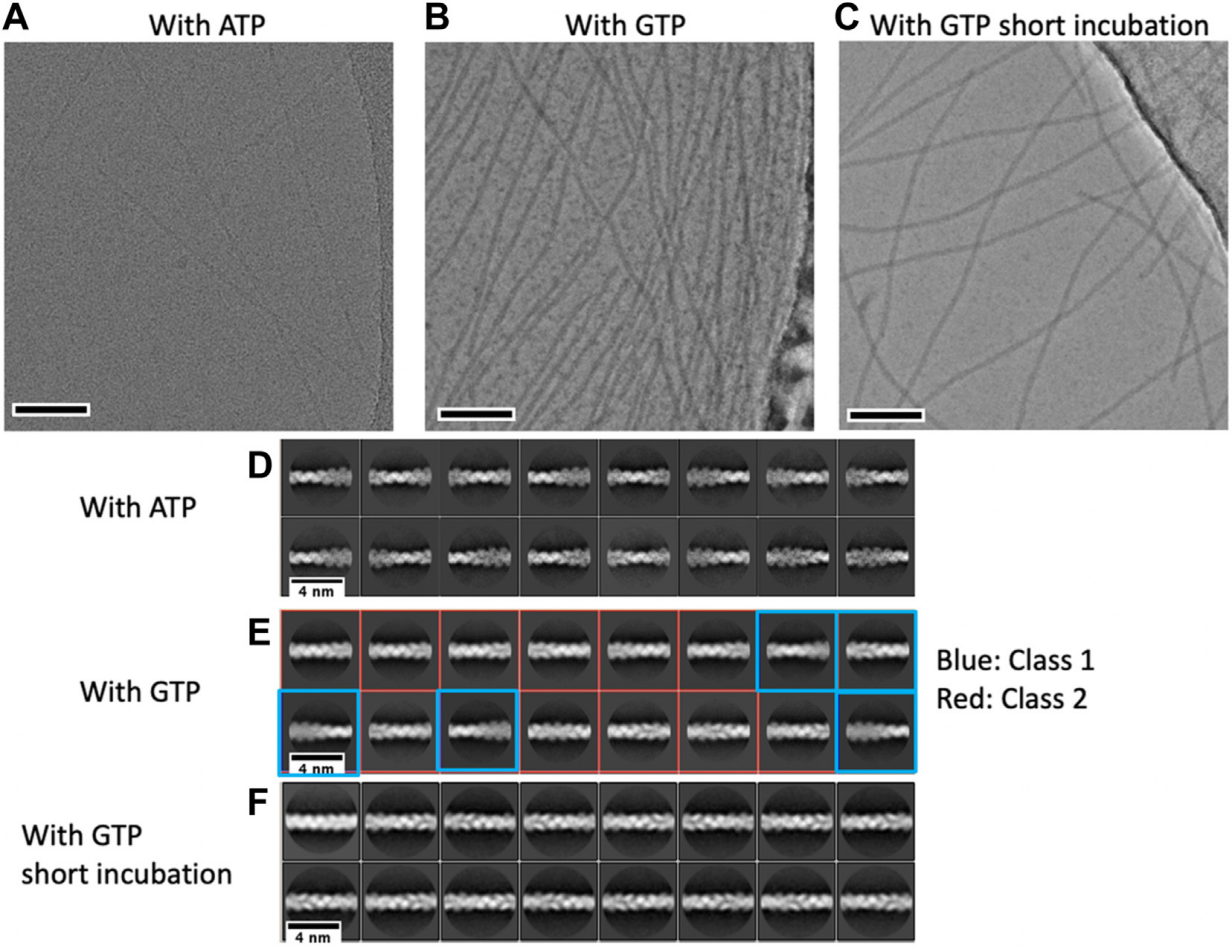
**Cryo-EM images of Cb-cParM filaments**. *A–C*, cryo-EM images of *Cb*-cParM filament formed with ATP, with GTP, or with GTP and short incubation time, respectively. Scale bars = 100 nm. *D–F*, averaged images after a 2D classification. The images with GTP were classified into two classes: class 1, similar to those with ATP; and class 2, similar to those with GTP and short incubation time.

We recorded CryoEM images of the *Cb*-cParM filaments after polymerization with ATP or GTP for 30 min. Filaments with similar diameters were observed under both conditions (Fig. 6, *A* and *B*). However, 2D classification showed substantially different averaged images (Fig. 6, *D* and *E*). The averaged images of *Cb*-cParM, polymerized with ATP, revealed obvious wide and narrow regions along the filament (Fig. 6*D*), indicating a possible strand crossover. The 2D classification images of *Cb*-cParM polymerized with GTP appeared to show a mixture of two states (Fig. 6*E*). Similar 2D class images were observed when non-hydrolysable nucleotide, AMPPNP was used (Fig. S13). One state was similar to *Cb*-cParM polymerized with ATP, and the other was distinct. We tried polymerization with GTP for a shorter incubation time (1 min, Fig. 6*C*), which yielded the latter of the mixed states. The averaged images from the short incubation time, showed a more uniform diameter along each filament (Fig. 6*F*).

**Figure 7:**
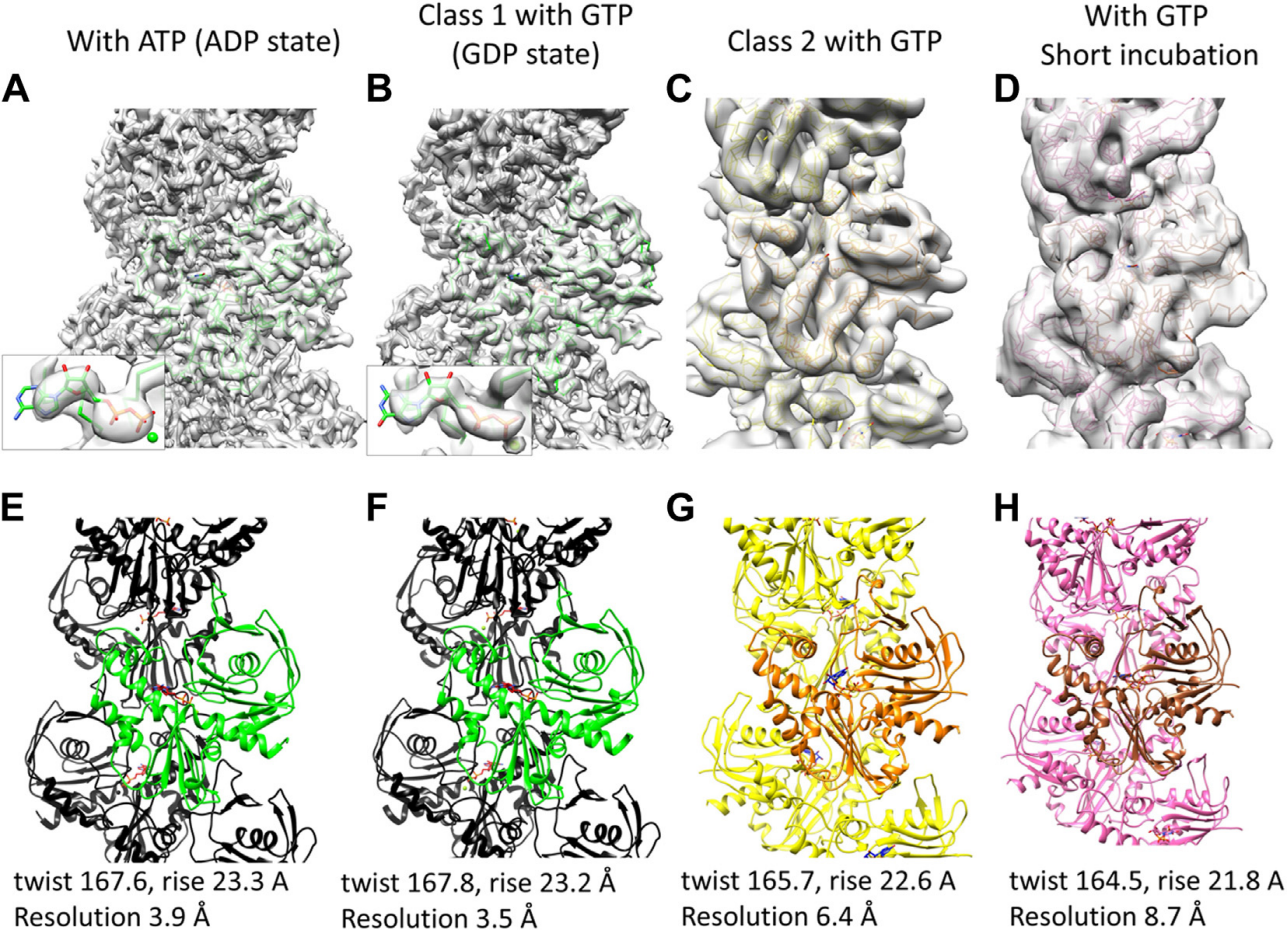
**Maps and *Cb*-cParM structural states with bound nucleotides.** The nucleotides are indicated in red and blue colors. *A* and *E*, with ATP. *B* and *F*, class 1 with GTP. *C* and *G*. class 2 with GTP. *D* and *H*. with GTP and short incubation time. The structures with ATP (*A* and *E*) were almost identical to those of class 1 with GTP (*B* and *F*). The gamma phosphate could not be observed in *A* and *B* (insets), showing that the binding nucleotides were ADP and GDP, respectively. The model for class 2 with GTP (*C* and *G*) and the model with GTP and a short incubation time (*D* and *H*) were similar to each other.

Four density maps were reconstructed for the three polymerization conditions (Fig. 7 and Table S5). One map corresponds to the first condition (ATP, 30 min, Fig. 7*A*), at 3.9 Å resolution. Two maps were reconstructed from the second condition (GTP, 30 min), corresponding to the two groups in the 2D classification, class 1 and class 2 (Figs. 6*E* and 7, *B* and *C*), and one map from the short GTP incubation (1 min, Fig. 7*D*). The map from class 1 at 3.5 Å resolution (GTP, 30 min, Fig. 7*B*) was very similar to that from the first condition (ATP, 30 min, Fig. 7*A*). The two models from the two maps (ATP 30 min, and class 1 with GTP 30 min), were identical up to the reliable resolution of the data, despite the difference in the bound nucleotides. In both maps, density due to gamma phosphate or inorganic phosphate around the nucleotide was not observed, indicating that the bound nucleotides had been hydrolyzed to ADP and GDP, respectively, and the phosphate was released. The maps from class 2 with GTP (30 min, Fig. 7*C*) and the third condition (GTP, 1 min, Fig. 7*D*) were similar to each other, although the resolution was limited, suggesting that they were in the state before the phosphate release. The helical twists for *Cb*-cParM and *Dh*-cParM1 protofilaments were different, ∼167° compared to ∼156°, respectively, with *Cb*-cParM being more similar to ParM-R1 (∼165°) (2).

### Crystal structure, rigid bodies, and domain movement

**Figure 8:**
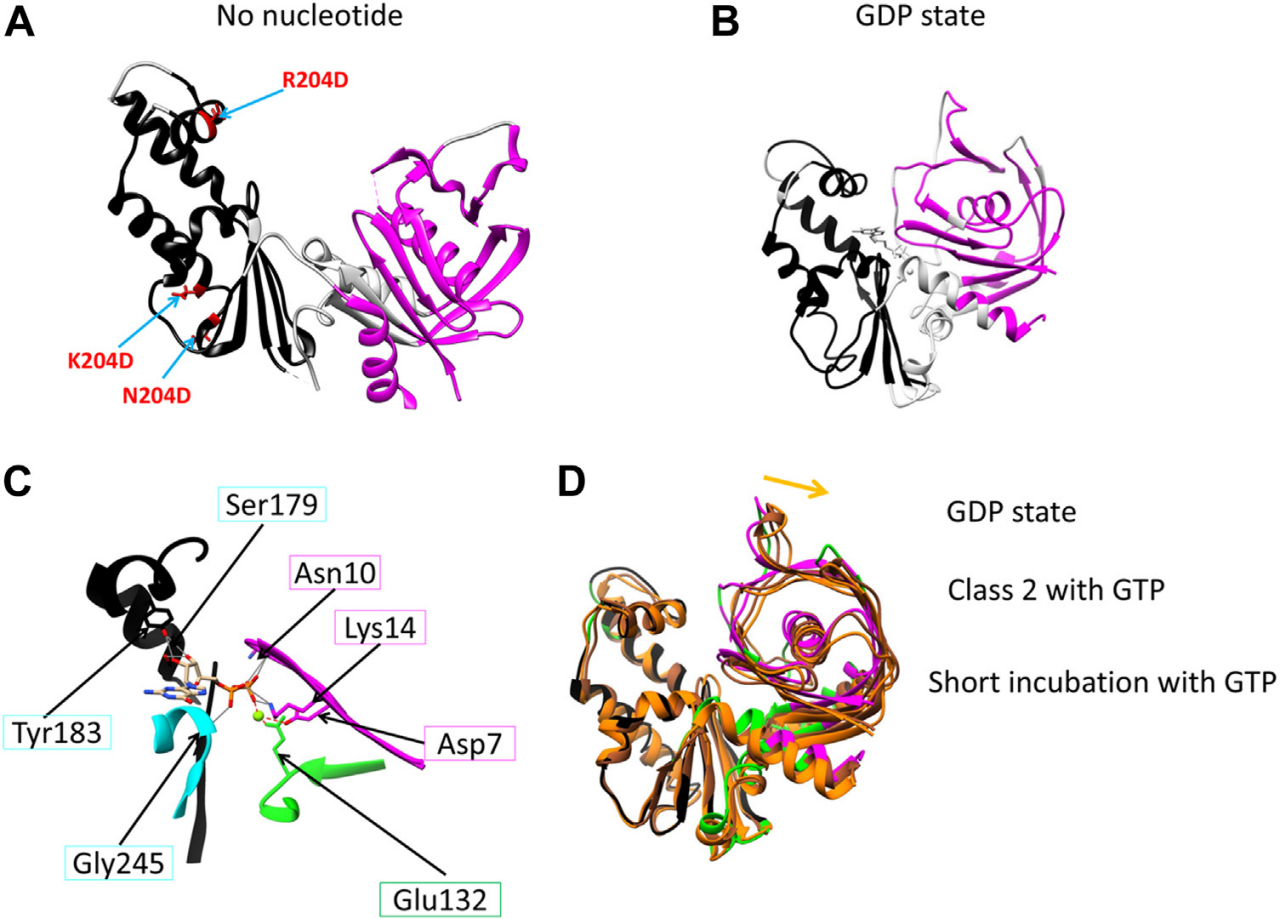
**Identification of rigid bodies in *Cb*-cParM.***A*, the crystal structure is without nucleotide. The side chains of R204D, K230D, and N234D are shown in *red* and are labeled. *B*, Cryo-EM structure with bound GDP (Fig. 7*B*). Two rigid bodies were identified by comparing these structures (ID rigid body, *black*, and OD rigid body, *magenta*). *C*, the bound GDP and Mg^2+^ connected the two rigid bodies (ID rigid body in *black*, OD rigid body in magenta, and the rest of the protein in *green*). Possible hydrogen bonds corresponding to GDP were determined using UCSF chimera [78] and presented as *gray lines* and possible salt bridges with the Mg^2+^ presented by *red dotted lines*, although additional hydrogen bonds *via* water molecules may exist. *D*, Models for the class 2 state with GTP (*orange*) and the short incubation time with GTP (*brown*) were aligned by the ID rigid body and superposed on the model with GDP (*green*, *black*, and *magenta*). The nucleotide-binding cleft was more open in the models for the class 2 with GTP and short incubation time with GTP relative to the GDP state. The orange arrow indicates the direction of domain movement in the open conformation.

We successfully obtained a crystal structure of the apo form of a mutant of *Cb*-cParM, without bound nucleotide (Fig. 8*A* and Table S6), in which the nucleotide-binding cleft was wide open. This mutant *Cb*-cParM was designed to prevent filament assembly *via* the substitution of three residues in the protomer subunit: subunit interface, R204D, K230D, and N234D (Fig. S14). We compared the high-resolution model built into the cryoEM map of the GDP state filament (Fig. 8*B*) with the crystal structure of the monomer without nucleotide. We identified two regions that remained almost identical in the large structural change between the models, like those in actin (45, 46). We named these regions, the inner domain (ID) rigid body and the outer domain (OD) rigid body (Fig. 8, *A* and *B*), after the actin rigid body nomenclature. In the GDP state, there were no direct interactions observed *via* hydrogen bonds or salt bridges between the ID and OD; instead, the GDP phosphates connected the two rigid bodies (Figs. 8*C* and S8*A*). This explains why the crystal structure without nucleotides adopts a wide open state, due to the lack of interactions between ID and OD. The *Cb*-cParM amino acids surrounding GDP are most similar to those of ParM-R1 when compared with the crystal structures of previously characterized ParMs (Fig. S8, *B*–*D*). The nucleotide-binding cleft was slightly more open in the models for class 2 with GTP and short incubation time with GTP, relative to the GDP state (Fig. 8*D*). In addition, the guanine moiety of the GDP did not form contacts or hydrogen bonds with the protein, explaining why *Cb*-cParM can utilize both GTP and ATP. The density of guanine and adenine moieties was relatively weak, indicating the flexibility of these parts due to their lack of interaction with the protein (Fig. 7, *A* and *B*).

### Filament structure

**Figure 9:**
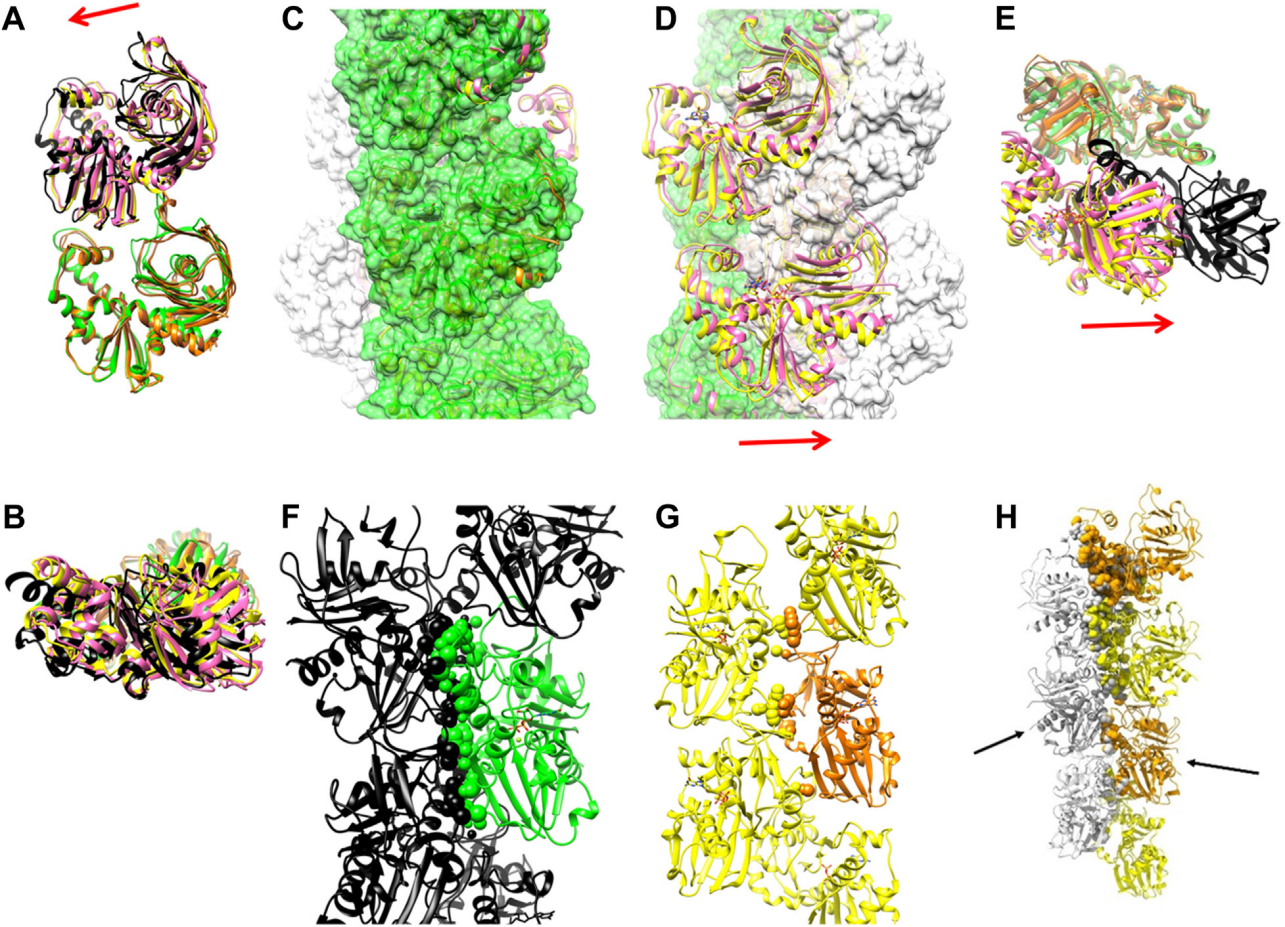
**Structural shift in *Cb*-cParM filament strands.***A* and *B*, two subunits in one strand (*A*: front view and *B*: top view). The ID rigid bodies of the lower subunit were aligned with each other. GDP state: *green* and *black*, class 2 with GTP: *orange* and *yellow*, short incubation with GTP: *brown* and *pink*. The interactions between the subunits appear similar in the three states except for the upper subunit position, which was slightly shifted in the GDP state compared to the other two states. The closure of the cleft in the GDP state may explain this difference because the closure of the cleft can push the upper subunit leftward. A *red arrow* indicates the direction of the shift of the upper subunit. *C–E*, relative positions of the two strands. *C*, the model with GDP is presented as a surface model in *green* and *light gray*. Ribbon models for class 2 with GTP (*orange* and *yellow*) and the short incubation time with GTP (*brown* and *pink*) are superposed. The ID rigid bodies of the center subunits are aligned with each other. *D*, 180° rotated view of C. The opposite strand position in the GDP state was completely different from that in the other two states (compare the *gray* surface to the cartoon), indicating a substantial strand movement after phosphate release. *E*, top view of two adjacent subunits in the different strands. GDP state: *green* and *black*, class 2 with GTP: *orange* and *yellow*, short incubation with GTP: *brown* and *pink*. The ID rigid bodies of the lower subunit (*green*, *orange*, and *brown*) were aligned with each other. A *red arrow* indicates the direction of the shift in *D* and *E* from the class 2 state with GTP to the GDP state. *F*, inter-strand interactions in the *Cb*-cParM filament model with GDP. Atoms from the center molecule (*green*) in contact with the other strand (*black*) are presented in space-filling representation. 176 contacts are observed. *G*, inter-strand interactions in the model for class 2 with GTP. 36 contacts were observed. *H*, the strands of the model with GDP (*light gray*) were replaced by the strands of the model for class 2 with GTP (*orange* and *yellow*). The strands were aligned by the molecules indicated by *black arrows*. The clashing atoms were presented in space-filling representation. The contacts and clashes were identified by ChimeraX (73).

The *Cb*-cParM filament reconstruction with ATP (30 min) was indistinguishable from that of class 1 with GTP (30 min). Therefore, we compared the three remaining filament models: (i) class 1 with GTP (30 min, GDP state), (ii) class 2 with GTP (30 min), and (iii) short incubation time with GTP (1 min). The intra-strand interactions between subunits were very similar in the three models (Fig. 9, *A* and *B*), except for a slight shift in the position of the adjacent subunit in the GDP state. This difference can be explained by the closure of the cleft in the GDP state, which can push the upper subunit leftwards. Despite the similarity within single stands, a large change was observed between strands in forming the filament structures. When the ID rigid bodies of one subunit of each state were aligned with each other, the positions of opposite strands were significantly different (Figs. 9, *C* and *D* and Movie S1), with a shift of ∼2.5 nm (Fig. 9, *D* and *E*). The GTP (30 min) class 2 state and GTP (1 min) are equivalent in their inter-strand orientations and have fewer cross-strand contacts than observed in the GDP state (Figs. 9, *F* and *G* and S15), suggesting stabilization of the filament bound to GDP. Thus, there are two dramatically different *Cb*-cParM filament conformations: one comprised of loosely associated strands (likely to be bound to GTP or GDP-Pi); and the second formed by a closer association of the strands bound to GDP (Figs. 9 and S15).

## Discussion

Orthologs of plasmid-encoded ParABS systems are found on bacterial chromosomes and function in chromosome segregation (20, 22). A replicated ParB-bound plasmid or chromosome is passed along a gradient of DNA-bound ParA, *via* an ATP hydrolysis mechanism, to the opposite pole of the cell to the site of replication (47). In contrast to this system, ParCMR segregation systems partition plasmids by forming elongating ParM filaments between two ParR-bound plasmids, pushing the plasmids to the two cell poles (48). ParCMR systems have yet to be implicated in chromosome segregation. Here, our findings demonstrate that some of the biochemical, biophysical, and structural properties of ParCMR homologs located on bacterial chromosomes are similar to those of plasmid-borne ParCMR systems.

Biochemically, both *Dh*- and *Cb*-cParMs polymerize to form filaments in a nucleotide-dependent manner (Figs. 5, *C*–*E*, 6, *A*–*C*, S5, and S6). Furthermore, protomers of the characterized cParMs (Figs. 3*D* and 8*D*) possess the characteristic ParM-closed beta-barrel domain (49), comprised of β-sheets wrapped around a helix, similarly as ParM-R1 (50), pSK41 plasmid ParM (14), BtParM (10), AlfA (18) and p*CB*H ParM (11). AlphaFold2-generated models (Fig. S1) indicate that the wider set of genome-borne ParMs also exhibit the beta-barrel subdomain. This demonstrates that the ParM systems borne on genomes bear resemblance and polymerization activities similar to plasmid-borne ParMs. Thus, cParMs likely have functions other than plasmid segregation, which are yet to be explored.

*Cb*-cParM polymerizes with ADP or GDP (Fig. 5*C*), whereas *Dh*-cParM is similar in this respect to other characterized ParMs (10, 11, 34). *Dh*-cParM polymerizes with ATP or GTP and is destabilized with ADP or GDP (Fig. S5*B*). It is likely that for *Dh*-cParM, polymerization with GDP was either short-lived or totally absent. The *Cb*-cParM filaments, polymerized with either di-phosphate nucleotide, were stable (Fig. S10). Phosphate release from *Cb*-cParM polymerized from ATP or GTP did not cause significant filament instability, as observed for other ParM systems, rather the filament underwent a significant structural change. More inter-strand contacts were observed in the GDP-bound state, providing stability after phosphate release (Figs. 9, *F* and *G* and S15). When the strands in the GDP model were replaced by the class 2 model with GTP, *in silico*, severe clashes were observed (Fig. 9*H*). Therefore, the small change in the strand structure (Fig. 9*A*) caused by a slight opening of the nucleotide-binding cleft (Fig. 8*D*) in the class 2 model with bound GTP prevents the formation of the close contacts observed in the model with GDP. After phosphate release, the strands adopt the GDP conformation allowing for new inter-strand contacts. Thus, phosphate release results in a large structural change in the filament.

The *Cb*-cParM cryoEM images showed obvious differences in the distribution of the nucleotide states between ATP and GTP at 30 min after initiation of polymerization. It implies either that phosphate release from the ADP-Pi state is faster than that from GDP-Pi state, or a faster rate of nucleotide hydrolysis, shortening time in the states of ADP-Pi and ATP in the nucleotide hydrolysis cycle. This is consistent with the faster phosphate release observed with ATP under steady-state conditions following the initial polymerization (Fig. 5*D*, gradient from 10 min).

We discovered that *Cb*-cParM filaments depolymerized by a different mechanism to filament instability following phosphate release. *Cb*-cParR acts as a depolymerization factor for the aged filament. We speculate that *Cb*-cParR recognizes the large structural change in *Cb*-cParM after phosphate release allowing it to adopt its role in depolymerization. This mechanism has parallels in the eukaryotic actin system, where cofilin senses the nucleotide status of the actin filament, resulting in depolymerization (51).

Since *Cb*-cParR also binds to *Cb-cparC*, it appears that *Cb*-cParR may have several potential roles. The DNA-bound *Cb*-cParR may be expected to bind to the ends of *Cb*-cParM filaments, by analogy to plasmid ParCMR systems. We speculate that the multiple copies of *Cb*-cParR bound to the repetitive sequences of *Cb*-c*parC* will track the *Cb*-cParM filament end. If the balance is towards polymerization then the growing *Cb*-cParM filament will exert a pushing force. However, if the *Cb*-cParR-induced depolymerization of the *Cb*-cParM filament is the dominant effect, then there will be a pulling force. *Cb*-cParR free of *Cb-parC* may act as a depolymerizing factor for non-productive *Cb*-cParM filaments (Fig. S12). By contrast, the *Dh*-cParM system acts like a classical ParM forming double stranded filaments in a nucleotide-dependent manner. If the *Dh*-cParR and *Dh*-*cparC* are both functional, then *Dh*-cParM polymerization may be expected to exert pushing forces on the chromosome. Total Internal Reflection Fluorescence (TIRF) microscopy studies are now needed to determine the activities of these cParCMR systems.

These two described bacterial genome-borne ParMR systems have been proven to be functional and active polymerization systems like those present on segregating plasmids. Additionally, we have discovered many other systems on genomes of bacteria which may, or may not, also possess plasmids (a representative selection is shown in Fig. 1). Whereas some cParMs such as *Nt*-cParMs are not conserved on the chromosome of *Natranaerobius* species*, Bt*- and *Mt*-cParMs, like *Dh*-cParM, are highly conserved in *Bacillus* and *Moorella* species, respectively (Table S1–S3 and Figs. S2–S4). The high sequence conservation within these bacterial species especially *Desulfitobacterium* species, together with the striking feature of the absence of plasmids in this bacterium (28), strengthens the hypothesis of indispensable functions other than plasmid partitioning. We cannot rule out the possibility that the cParMs encoded on genomes contribute to plasmid segregation when plasmids are present. However, in the case of the *Cb*-cParCMR cassette, a functional (*Cb*-cParR binding) *parC* is borne on the genome, implying this cassette most probably is involved in a different role than plasmid segregation. (21, 22, 52, 53). ParA and ParB genes are also found on the genomes, or on related genomes (*C. botulinum* NZ_CP013247.1), which harbor the cParMR systems identified here (Fig. 1), indicating that cParCMR is not a replacement segregating system for ParABS. However, the presence of genome-borne ParCMR systems paves the way for further studies to experimentally explore whether these systems supplement the chromosome-segregating ParABS system, or whether they play other undiscovered roles.

## Experimental proedures

### Bioinformatics analysis and identification of ParMR operons

A bioinformatics sequence database search (NCBI and Uniprot databases) was performed on bacteria genomes to identify the presence of the ParMR loci using the conserved actin superfamily fold, including the nucleotide-binding region of the ParM sequence (52). Next, ParR was searched for either upstream or downstream of the ParM gene (53). Unlike ParMs, which inherit the nucleotide-binding region allowing for easy identification, ParR does not possess such a characteristic key motif. Finally, a DELTA-BLAST search was used to locate potentially overlooked ParM and ParR sequences.

For the synteny analysis, *Desulfitobacterium* genomes with conserved ParM sequences were selected through blast searches (https://blast.ncbi.nlm.nih.gov/Blast.cgi?PAGE=Proteins) and the genome sequences were downloaded from the NCBI genome database (https://www.ncbi.nlm.nih.gov/datasets/genome/). Genetic loci containing ParMs were collected through an easy-search command of mmSeqs2 (54). To depict synthetically conserved genes, roughly 25 kb regions surrounding cParM genes were trimmed and depicted by clinker (55). Multiple sequence alignments for cParMs identified in *Desulfitobacterium* genomes were conducted based on the MUSCLE algorithm implemented in Jalview (56, 57). For the statistical analysis of cParM conservation in *Desulfitobacterium* genomes, sequence alignments were conducted taking CDSs of one genome as queries and CDSs from another genome as a database through easy-search command of mmSeqs2 (54). The output was parsed, and only the top hits for individual sequences were chosen to draw histograms by pandas.DataFrame.plot.hist on Pandas v.1.5.3 (58) and Python v.3.11. (https://www.python.org/downloads/release/python-3111/). All the histograms were depicted with 50 bins spread across 0 to 100% identities. Vertical axes indicate the number of genes categorized in individual bins.

### Protein expression and purification

Genes for cParMs and cParRs were synthesized and cloned into pSY5 and pSNAP vectors, respectively, encoding an 8-histidine tag followed by a human rhinovirus HRV 3C protease cleavage site. Plasmids were transformed into BL21 (DE3) cells grown to OD_600_ ∼ 0.8, and protein expression was induced with 0.2 to 1.0 mM IPTG overnight at 15 °C. The cultures were then centrifuged at 4000*g* and the cell pellets were resuspended in 50 mM Tris–HCl, pH 8.0, 500 mM NaCl, 20 mM imidazole, 5% glycerol, 0.5 mg/ml lysozyme, 0.1% Triton-X, and protease inhibitor tablets (1 per 2 L culture, Roche) and lysed *via* sonication. The cell lysate was then clarified by centrifugation at 30,000*g* for 30 min and filtered through a 0.45 μm membrane. The filtered supernatant was loaded onto a HisTrap FF 5 ml (GE Healthcare) pre-equilibrated with 50 mM Tris-HCl (pH 8.0) containing 500 mM NaCl and 20 mM imidazole. Following a washing step, human rhinovirus HRV 3C protease (5 mg/ml) was loaded in the same buffer for cleavage of tagged proteins (12 h at 4 °C). The cleaved protein was then eluted with washing buffer, pooled, concentrated, and subjected to size-exclusion chromatography (Superdex 75 pg, GE Healthcare) in 40 mM HEPES pH 7.5, 150 mM KCl, 2 mM MgCl_2_, and 1 mM DTT. Fractions were checked for purity *via* SDS–PAGE, and the pure fractions were pooled and concentrated to between 5 and 10 mg/ml, as determined by UV absorbance at 280 nm using an estimated A_280_ value calculated using PROTEINCALCULATOR v3.4 (http://protcalc.sourceforge.net).

### Electrophoretic mobility shift assay

The reaction mixture (10 μl) containing 20 nM to 20 μM of cParR in 25 mM HEPES-HCl (pH 7.5), 300 mM KCl, 1 mM MgCl_2_, 0.5 mM DTT, 1 mg/ml bovine serum albumin, 0.1 μg/μl sonicated salmon sperm DNA, and 5% glycerol was mixed at 25 °C for 10 min, followed by the addition of 20 nM 5′-FAM-labeled *cparC* DNA fragments and further incubation for 20 min. The polyacrylamide gels were prerun at 150 V for 1 h. After incubation, reactions were analyzed by electrophoresis on a 1 × TBE (pH 7.5), 4% polyacrylamide gel in 1 × TBE running buffer (0.89 M Tris-base, 0.89 M boric acid, 0.02 M EDTA, pH 8.3) at 150 V for 1 h. Gels were scanned using a Pharos FX Plus Molecular Imager (Bio-Rad) with an attached external laser.

## Polymerization assays

Assembly and disassembly of cParMs at 24 °C was followed by light scattering at 90° using a PerkinElmer LS 55 spectrometer for extended time measurements (initial delay time due to mixing by hand ∼ 10–50 s) or a BioLogic stopped-flow machine to observe the early polymerization phase (initial delay time ∼ 3 ms), monitored at 600 nm. Assembly was initiated by the addition of 2 mM nucleotide at 24 °C in 20 mM Hepes pH 7.5, 350 mM KCl, 2 mM MgCl_2_ (*Cb*-cParM), and 40 mM HEPES, pH 7.5, 400 mM KCl, 1 mM MgCl_2_ (*Dh*-cParM).

### Pi release assay

*Cb*-cParM protein (10 μM) was mixed with the appropriate nucleotide in the same buffer as the light scattering conditions and incubated according to the time course. The reaction was stopped by adding an equal volume of ice-chilled 0.4 M perchloric acid (PCA). The reaction mixture was then centrifuged at 1700*g* for 1 min. Equal volumes of supernatant and Malachite Green reagent were mixed and incubated at 25  °C for 25 min (59). Absorbance was recorded at 650 nm with a Hitachi U-3010 spectrophotometer.

### Sedimentation assay

The polymerization of *Dh*-cParM (20 μM) with different nucleotides was monitored after the addition of 5 mM nucleotide (ATP, ADP, GTP, or GDP) in 40 mM HEPES (pH 7.5), 300 mM KCl, 2 mM MgCl_2_, and 0.5 mM DTT buffer at 24  °C for 30 min. Samples were centrifuged at 279,000*g* for 20 min. Pellets were resuspended in the same volume of the buffer as the reaction mixture. Supernatant and pellet fractions were analyzed through SDS-PAGE.

To investigate the critical concentrations for polymerization, polymerization of different concentrations of *Cb*-cParM (0.5–4.5 μM) was initiated by the addition of 5 mM nucleotide (ATP, ADP, GTP, or GDP) in 40 mM HEPES (pH 7.5), 150 mM KCl, 2 mM MgCl_2_, and 0.5 mM DTT at 24  °C for 30 min. Samples were centrifuged at 279,000*g* for 20 min and pellets were resuspended in the same volume as the reaction. Concentrations of *Cb*-cParM in the pellet and whole fractions were estimated *via* SDS–PAGE and gel images were scanned by Printgraph AE-6933FXCF (ATTO) and analyzed using ImageJ software. The standard plot was determined with known concentrations of *Cb*-cParM. The critical concentration marks the concentration at which the linear plot intersects with the x-axis (60).

To investigate the effects of *Cb*-cParR on *Cb*-cParM, polymerization of *Cb*-cParM (20 μM) with and without *Cb*-cParR (20 μM) and *Cb-cparC* (20 μM) was initiated by the addition of 5 mM nucleotide (ATP, ADP, GTP, and GDP) in 40 mM HEPES (pH 7.5), 350 mM KCl, 2 mM MgCl_2_, and 0.5 mM DTT at 24  °C for 30 min. Samples were centrifuged at 279,000*g* for 20 min and pellets were resuspended in the same volume as the reaction and the concentrations of *Cb*-cParM in the supernatant were estimated *via* SDS–PAGE.

## Crystallography

A *Cb*-cParM mutant was constructed with three mutations (R204D, K230D, and N234D) to prevent polymerization and allow for crystallization. Purified protein was subjected to crystallization trials by mixing and incubating 5 mg/ml *Cb*-cParM mutant and 1 mM AMPPNP on ice for 1 h. Via the hanging drop vapor diffusion method crystals were grown in 0.5 μl of protein/AMPPNP and 1 μl of mother liquor (0.2 M ammonium chloride, 22% (w/v) PEG 3350) at 288 K. X-ray diffraction data were collected on a RAYONIX MX-300 HS CCD detector on beamline TPS 05A (NSRRC, Taiwan, ROC) controlled by BLU-ICE (version 5.1) at λ = 1.0 Å. Indexing, scaling, and merging of data were performed using HKL2000 (version 715) (61). Molecular replacement using the protomer built into the 3.5 Å cryoEM density map was carried out in the Phaser (62) after splitting the structure into the two domains. Model building was carried out in Coot (63) and refinement in PHENIX (62). Data collection and final refinement statistics are summarized in Table S6. Although *Cb*-cParM was crystallized in the presence of AMPPNP, the resultant structure did not contain nucleotide.

### Cryo-electron microscopy of *Dh*-cParM

ParM protein (20 μM) was polymerized with 5 mM ATP in 40 mM HEPES, pH 7.5, 400 mM KCl, and 1 mM MgCl_2_ buffer at room temperature for 20 to 30 min. The mixture (2.5 μl) was applied to previously glow discharged R 1.2/1.3 Molybdenum 200 grids with a holey carbon support film (Quantifoil). Grids were quickly transferred to an EM GP Leica for blotting for 1.5 to 2.5 s at 90% humidity and flash-frozen in liquid ethane cooled by liquid nitrogen. Grids were stored under liquid temperatures and screened on a Tecnai G2 Polara (FEI, Nagoya University) operated at 300 kV at a minimal dose system. Final cryoEM data were collected on a Titan Krios (FEI, Osaka University) equipped with FEG operated at 300 kV and a minimal dose system. Imaging was done using the EPU software (FEI) attached to the Titan Krios. Images were recorded at a nominal magnification of 75,000, objective aperture of 100 μm, actual defocus range between −1.5 to −2.5 μm with a dose rate of 45 e^−^/Å^2^/s, and exposure time of 1 second with three image acquisitions per hole. Images were recorded using a Falcon II detector (FEI) at a pixel size of 1.1 Å/pixel and a frame rate of 17 frames/s. The first two frames were discarded and successive seven frames were saved for image processing.

### Image processing of *Dh*-cParM

About 2200 to 2500 images were collected from different microscope sessions and processed using RELION 2.0/3.0/4.0 (35, 64, 65). Frames were motion-corrected with MotionCorr (66) and CTF estimation was performed with Gctf (67). Micrographs with good observed CTF were selected for further processing. Using e2helixboxer, filaments were manually picked and extracted at a box size of 400 × 400 pixels in RELION 2.0/3.0/4.0. Particles from 2D classes displaying clear secondary structure elements were selected. Initial 3D reference models were prepared using conventional helical reconstruction from negative stain images using EOS software (68). A 3D refinement was performed with a low pass filter of 40 Å and helical symmetry search without solvent flattening. Converged helical symmetry was obtained and additional 3D refinement was performed with determined helical parameters with solvent flattening. Particle polishing, movie refinement without CTF refinement, and final 3D refinement were performed using the previous 3D refinement as the initial model producing a 4.0 Å map of *Dh*-cParM converged to a rise of 24.5 Å and twist of 156.03°. With post-processing using solvent flattening and a soft mask, final resolutions were attained for the ParM filaments. Handedness was determined by the handedness of the alpha helices observed in a single strand of the high-resolution filament structure.

### *Cb*-cParM cryo-electron microscopy

*Cb*-cParM (0.7 mg/ml) was polymerized in 20 mM HEPES-HCl pH 7.5 containing 250 mM KCl, 1.7 mM MgCl_2_, 3 mM GTP, or ATP. The mixed solution was incubated for 30 min or 1 min at 25 °C. R1.2/1.3 Mo400 grids (Quantifoil) were glow discharged and used within an hour. The reaction mixture (2.5 μl) was applied on the glow discharged grids, blotted on the EM GP (Leica), and vitrified by plunging in liquid ethane cooled by liquid nitrogen. Frozen grids were kept under liquid nitrogen for no more than 1 week before imaging. To screen for optimum conditions for cryoEM imaging, the grids were manually observed in a Tecnai G2 Polara (FEI) cryo transmission electron microscope (at Nagoya University) equipped with a field emission gun operated at 300 kV and a minimal dose system. Images were captured at a nominal magnification of ×,115,000 with an underfocus ranging from 1.5 to 3.5 μm and by subjecting the sample to a 2 s exposure time corresponding to an electron dose of ∼30 electrons per Å^2^. Images were recorded on a GATAN US4000 CCD camera using an energy filter operated between 10 and 15 eV, with each pixel representing 1.8 Å at the specimen level at exposure settings. Samples were imaged using a Titan Krios microscope operated at 300 kV installed with EPU software (Thermo Fisher) at Osaka University. The imaging parameters were nominal magnification of 75,000 and 91,000, actual defocus of 1.0 to 3.0 μm, dose rate 45 e^−^/Å^2^/s, exposure time 1 s, and three image acquisitions per hole. The images with ATP were recorded with a Falcon II/III detector (FEI) (Thermo Fisher) at a pixel size of 0.87 Å/pixel with an objective aperture 100 μm, while the images with GTP and 30 min incubation time were recorded with a Falcon II/III detector (FEI) (Thermo Fisher) at a pixel size of 0.87 Å/pixel with an objective aperture 100 μm. The images with GTP and 1 min incubation were recorded with a Falcon III (Thermo Fisher) at a pixel size of 0.87 Å/pixel with a phase plate.

### *Cb*-cParM image processing

From *Cb*-cParM samples with ATP, 2778 images were collected. Image processing was performed using RELION 3.1 (64, 69) software. After motion correction and contrast transfer function (CTF) estimation with CTFFIND-4.1 (70), images were selected for further image processing. Filaments were manually picked with e2helixboxer, after which particles were extracted at a box size of 384 × 384 or 400 × 400 pixels. After 2D classification, 36,762 particles were selected. The initial 3D reference was prepared using conventional helical reconstruction using EOS (68). Helical symmetry converged to 167.6° twist/22.3 Å rise along the left-handed helix, and the resolution reached 3.9 Å. With *Cb*-cParM GTP and 30 min incubation time, 1398 images were collected. After motion correction and CTF estimation, 152,490 particles were extracted. We categorized 2D averaged images into two by visual inspection. The first category (class 1) contained 40,599 particles and the helical symmetry converged to 167.8 ° twist/22.3 Å at 3.5 Å resolution, while the second (class 2) contained 70,754 particles and helical symmetry converged to 165.7° twist/21.7 Å rise at 6.5 Å resolution. For the map with GTP and 1 min incubation time, 2772 images were collected, and 153,326 particles were used for the final reconstruction at 8.6 Å resolution.

### Model building

The initial atomic model of *Cb*-cParM with GDP was constructed by homology modeling using Rosetta3 with the p*CB*H ParM model (11) (6IZV) as a template. The resulting model was iteratively refined using COOT (71), molecular dynamics flexible fitting (MDFF, using ISOLDE (72), an extension of ChimeraX (73), and Phenix (74). GDP in the final GDP model was replaced by ADP to give the initial model with ADP, which was then refined using the same procedures. The final GDP model was also used as the initial model for the lower resolution structures (PDBIDs 7X55 and 7X59), which were fitted into the map by MDFF with adaptive distance restraints for the two rigid bodies using ISOLDE (72). The resultant model was refined using COOT and Phenix.

For *Dh*-cParM1, we used AlphaFold2 software (30) to generate initial models. The model was refined with the cryoEM density map using ISOLDE software (72), and the Rosetta program (75) and subsequently refined using PHENIX real space to produce the protomer structures.

### Rigid body search

For *Cb*-cParM, the model with GDP and the crystal structure without nucleotides were aligned with each other to maximize the number of Cα with less than 0.7 Å deviation between the two models. The resultant residues with less than 0.7 Å deviation were considered as the rigid body (46). Two rigid bodies were identified (Fig. 8).

## Data availability

The filaments coordinates from this publication have been deposited in the Protein Data Bank (PDB, https://www.rcsb.org/) under accession codes are 8X1I (*Dh*-cParM1-ADP) and for *Cb*-cParM: 7X54, 7X56, 7X59 and 7X55 for ADP state, GDP state, the second class with GTP, and the short incubation time with GTP, respectively. The corresponding EM maps have been deposited in the Electron Microscopy Data Bank (EMDB, https://www.ebi.ac.uk/emdb/) are EMD-37996 (*Dh*-cParM1) and EMD-33007, 33009, 33012 and 33008 (*Cb*-cParM). The apo *Cb*-cParM X-ray structure is deposited in the PDB (7X3H). All other data are available from the corresponding author upon reasonable request.

## Supporting information

This article contains supporting information.

## Conflict of interest

The authors declare that they have no conflicts of interest with the contents of this article.
