# Supplementary material for: Bacterial genome-encoded ParMs[image]
Source: J Biol Chem. 2025 Jun 9;301(7):110351. doi: 10.1016/j.jbc.2025.110351 (PMC12270681; doi:10.1016/j.jbc.2025.110351)
Supplement: Supplementary-Data-3 [file mmc1.docx]

**Supplementary Data**

**
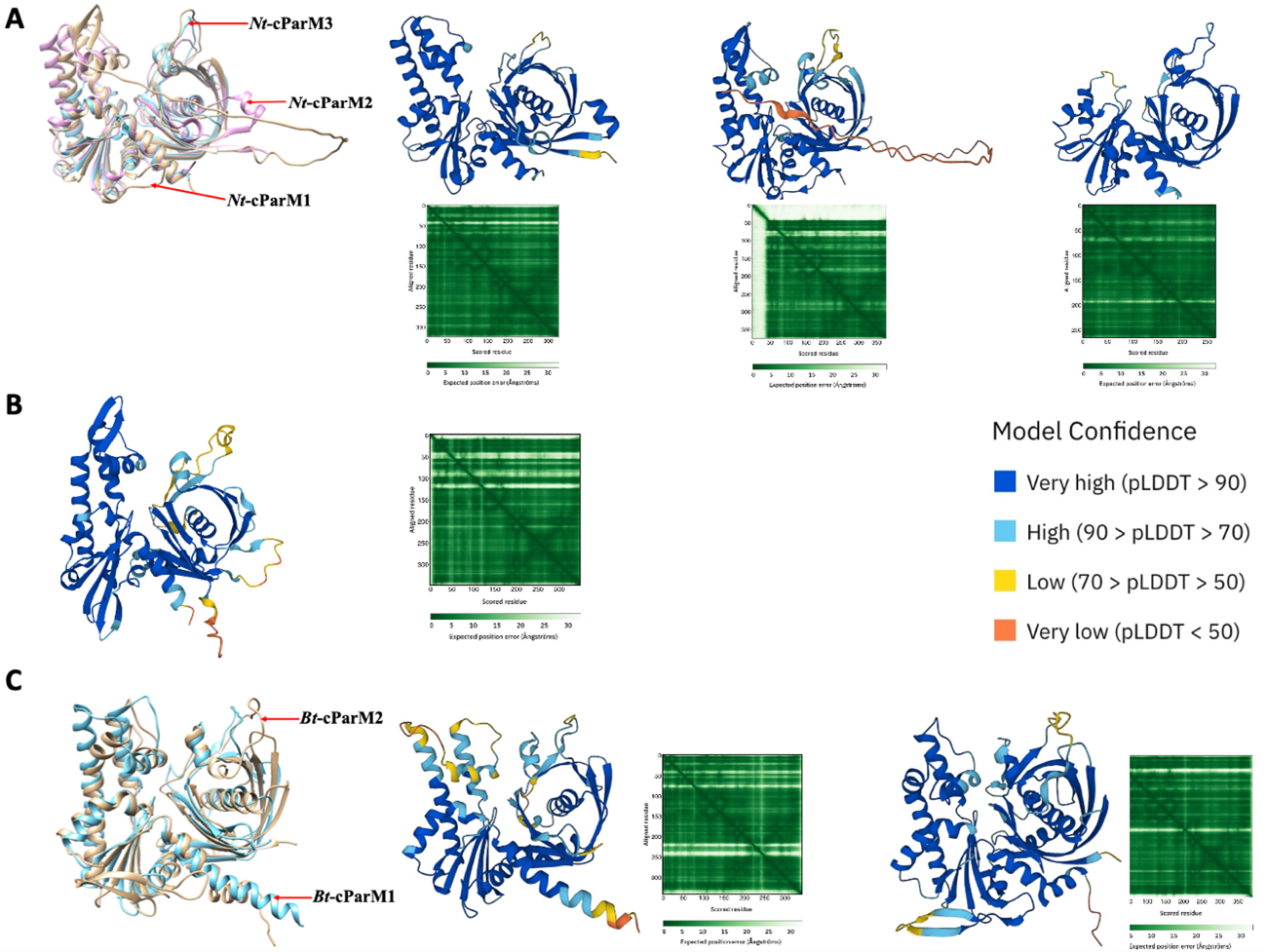
**

**Figure S1: AF2 models of selected cParMs. (A)** *Nt*-cParMs – *Nt*-cParM1 (WP_148206872.1) (pink), *Nt*-cParM2 (WP_012448769.1) (brown) and *Nt*-cParM3 (WP_012446843.1) (blue) encoded on the *Natranaerobius thermophilus* JW/NM-WN-LF strain chromosome **(B**) A single copy of *Mt*-cParM (WP_011391888.1) encoded on the chromosome from *Moorella thermoacetica* strain 39073-HH **(C)**  *Bt*-cParMs - *Bt*-cParM1 (WP_001968526.1) (blue) and *Bt*-cParM2 (WP_000025611.1) (brown) encoded on the *Bacillus tropicus* strain FDAARGOS_920 chromosome. Each individual AF2 model is shown with different colours based on the pLDDT model confidence score. For pLTTD plots, AlphaFold2 produces a per-residue model confidence score (pLDDT) between 0 and 100. Some regions below 50 pLDDT may be unstructured in isolation. Below or adjacent the models are the respective Predicted Aligned Error (PAE) plots of the various regions of the models. Each 2D plot square shade of green indicates the expected distance error in Ångströms (Å) for a pair of residues. A dark green tile designates a good prediction or low error whereas a light green tile indicates poor prediction or high error.

**Figure S2. cParMR clusters of *Desulfitobacterium* bacteria.** cParMR clusters and surrounding genes are aligned and depicted schematically for *Desulfitobacterium* genus by clinker [2]. Individual genes are represented as arrows labelled with the corresponding accession IDs. Homologous sequences above the identities of 30 % are connected by bands of corresponding colors for individual families. cParM (*red*) and cParR (*green*) are colored individually. For some strains, there are more than one cParMR clusters on the same genome.

**Figure S3. Multiple sequence alignment of cParMs identified in *Desulfitobacterium* genomes.** cParM sequences collected from *Desulfitobacterium* genomes were aligned through MUSCLE algorithm on Jalview [3, 4]. Conserved residues are colored according to the clustalx color scheme. Individual entries are labelled with accession NCBI IDs. The top five sequences refer to the highly conserved cParM1s.

**
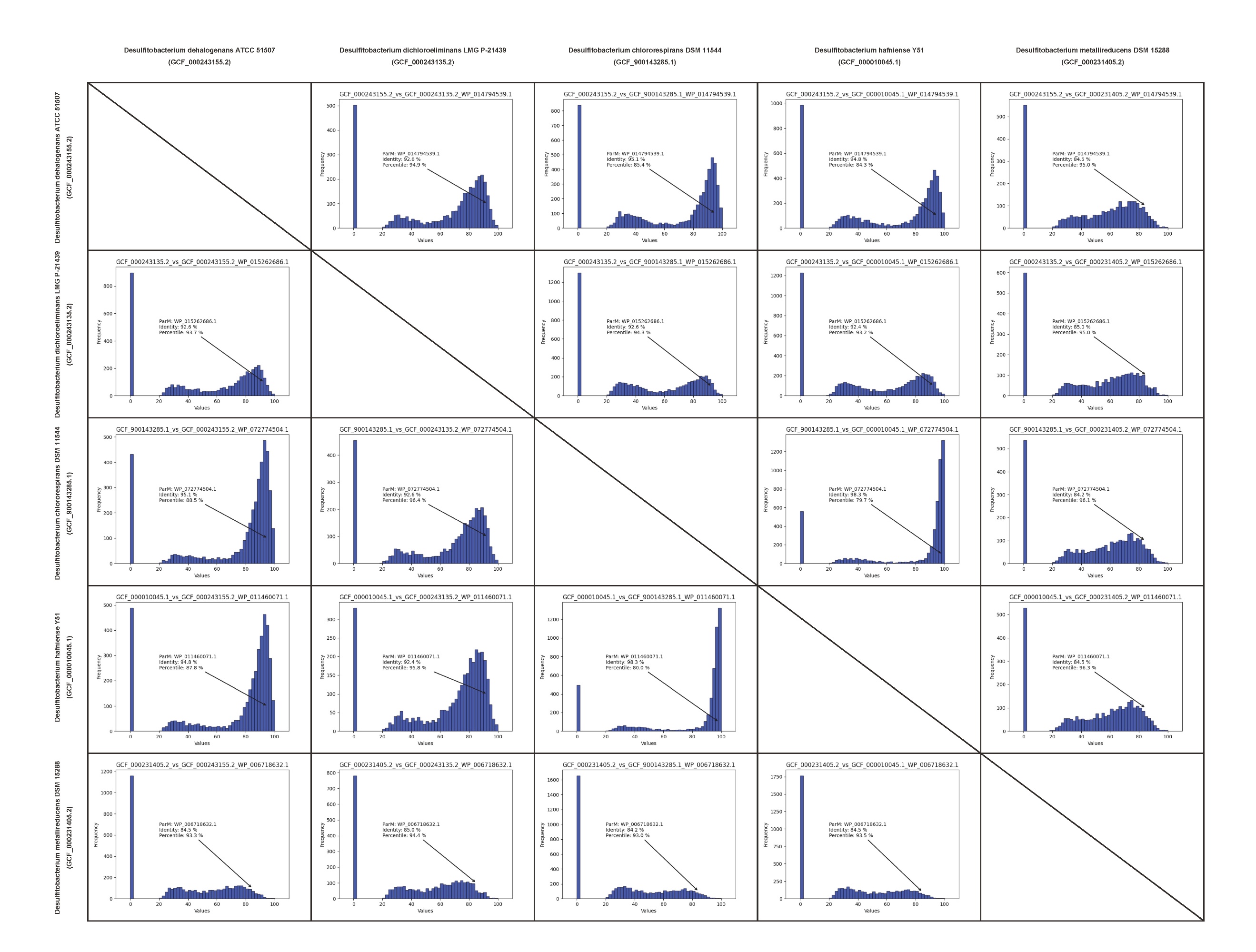
**

**Figure S4. Histograms of identities of protein Coding Sequences (CDSs).** Histograms of best identities of query CDS (row) against target CDS (column) are depicted based on pairwise sequence alignments of individual CDSs generated by mmSeqs2 [5]. Corresponding bins for cParM homologs, conserved amongst *Desulfitobacterium* genus, are indicated by arrows with labelled accession IDs, identities, and percentiles ranked from the lower end. The cParM homologs are well conserved amongst these five strains of the *Desulfitobacterium* genus. Histograms of coding sequence identities show that the identity of cParM between two species is consistently high compared to the identities of other proteins in the same species pair. The percentage identity of cParMs is greater than 84% in all strains. Here, the CDS refers to the sequence of amino acids in the protein.

**
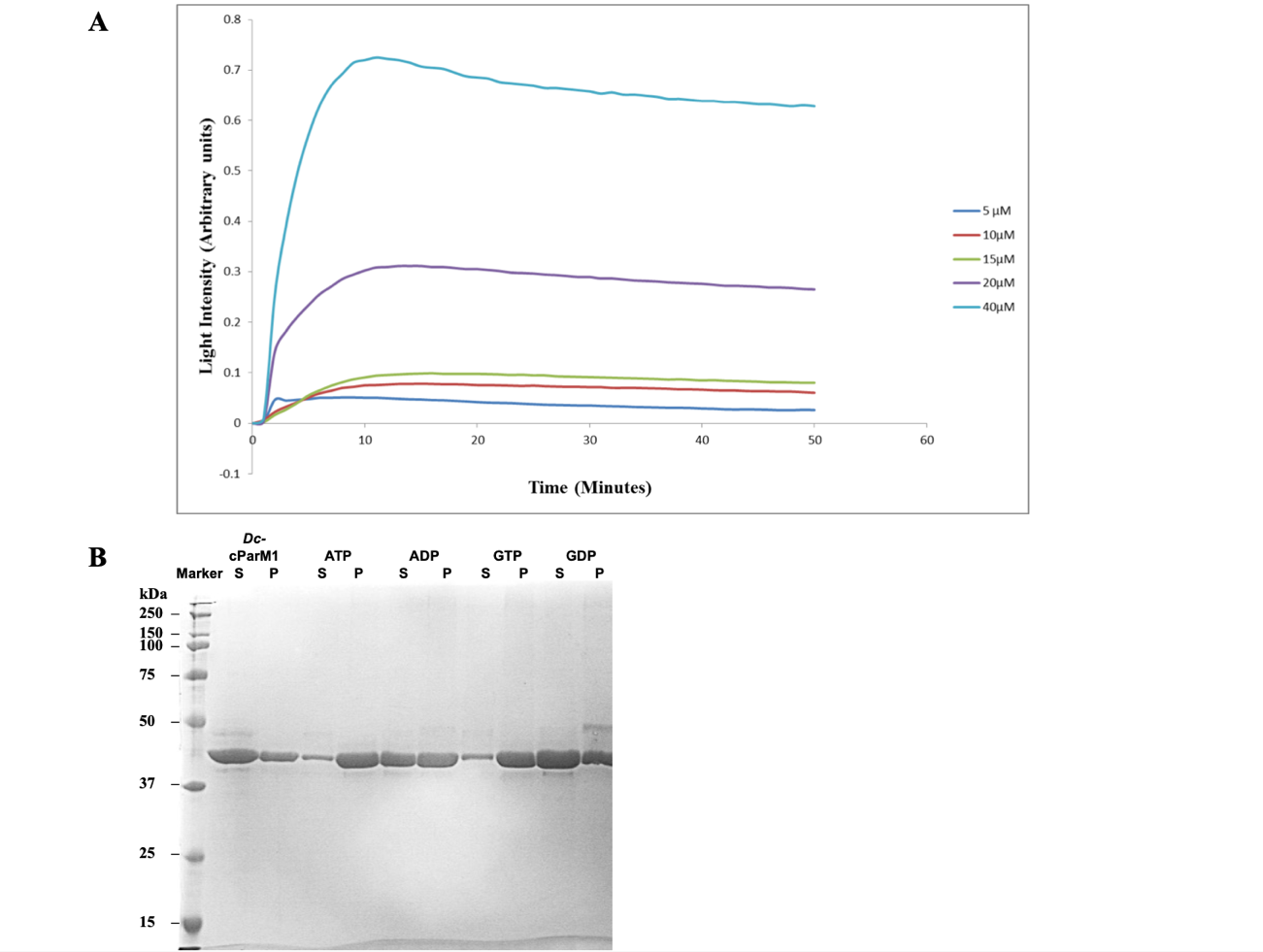
**

**Figure S5: *Dh*-cParM1 polymerization with different nucleotides. (A)** Different *Dh*-cParM1 concentrations polymerized with 5 mM ATP monitored by light scattering. All readings were taken at a wavelength of 600 nm and in a potassium chloride buffer (300 mM potassium chloride, 40 mM HEPES, pH 7.5, 1 mM MgCl_2_) at 25 °C **(B)** An SDS-PAGE gel of the sedimentation assay indicating the polymerization of 20 µM *Dh*-cParM1 with/without 5 mM of different nucleotides. Key: Marker, the standard protein marker; *Dh*-cParM1, protein without nucleotide; other lanes indicate the nucleotide used; S, soluble fraction; P, pellet fraction.


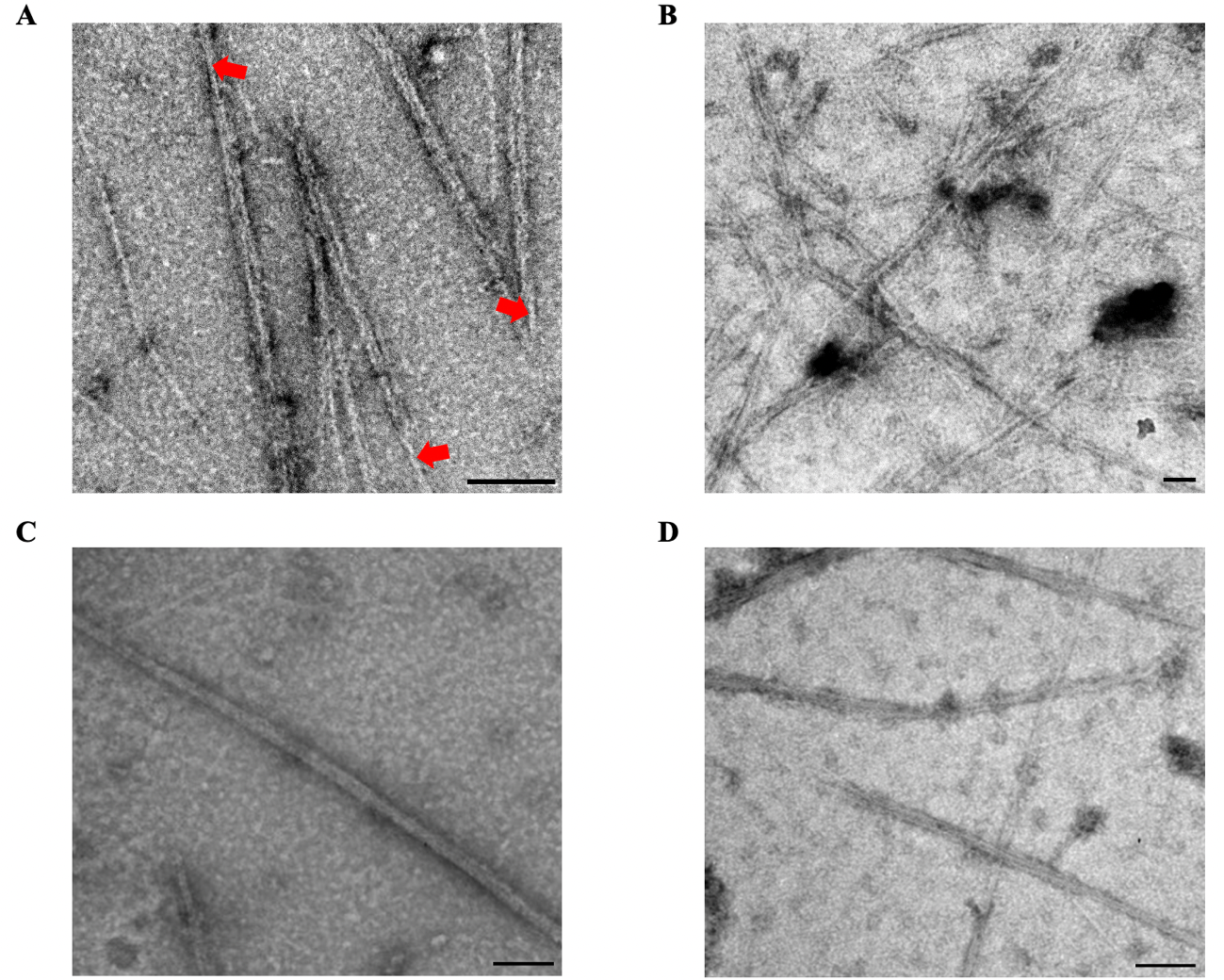


**Figure S6: *Dh*-cParM1 confirmation of filament formation in negatively stained samples.** 20 µM *Dh*-cParM1 polymerized with 5 mM of different nucleotides. Micrographs displaying the two types of *Dh*-cParM1 filament morphologies formed by (**A**) ATP (**B**) GTP (**C** ) AMPPNP (**D**) ATP-γ-S. The widths of coupled filaments narrow at the ends of the filament as indicated by the red arrow heads in **(A)**. All micrographs were prepared in potassium chloride buffer (300 mM potassium chloride, 40 mM HEPES, pH 7.5, 1 mM MgCl_2_) incubated at 25 °C. Scalebar = 100 nm.

**
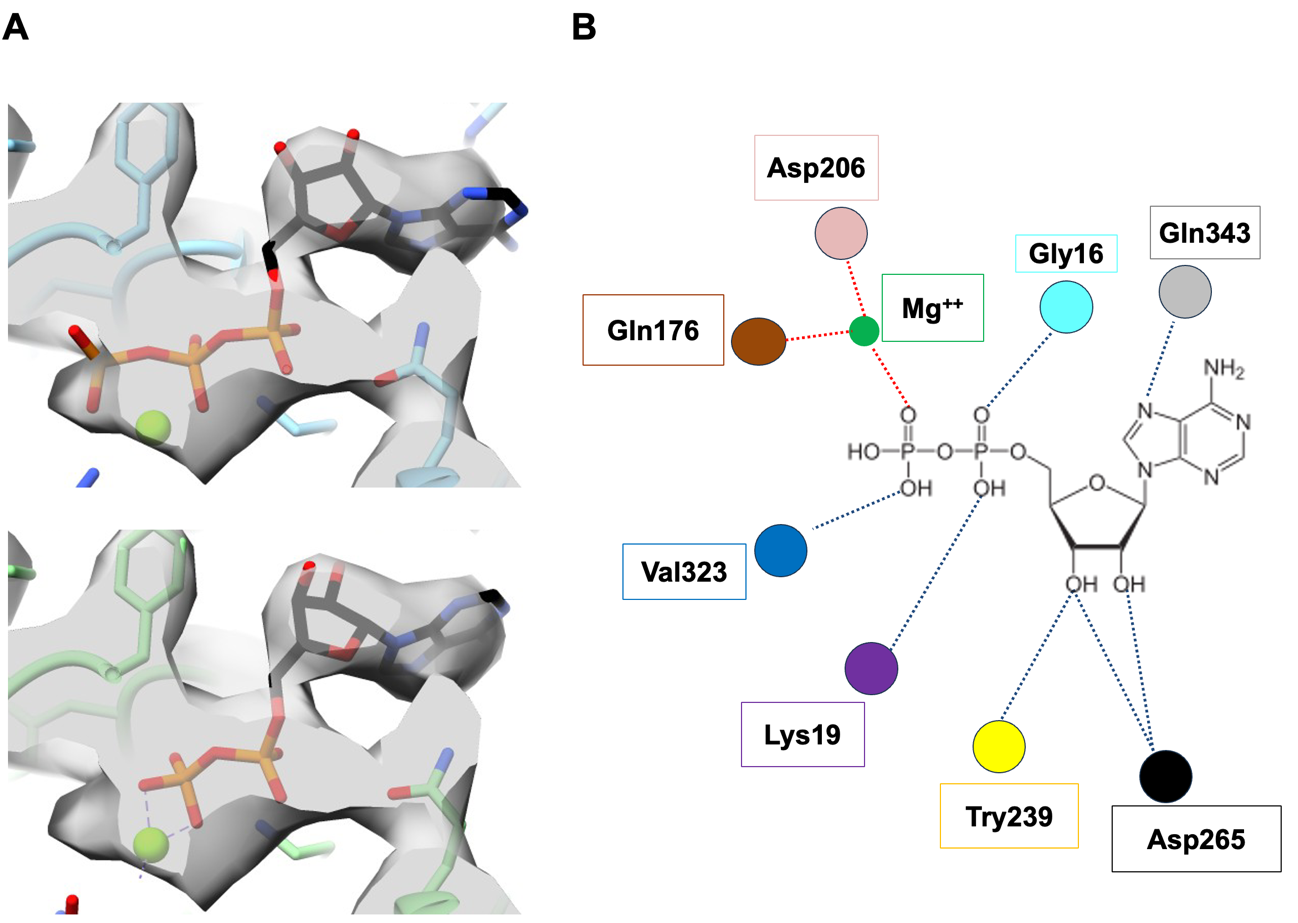
**

**Figure S7: Type of nucleotide in *Dh-cParM1* and its surrounding recognition amino acids. (A)** ATP (upper) and ADP (lower) fit into the cryoEM density map with ADP fitting slightly better. However, the difference is not pronounced due to resolution limitation. **(B)** A schematic diagram displaying the nucleotide recognition amino acids surrounding the ADP bound state of *Dh*-cParM1 in the filament.

**
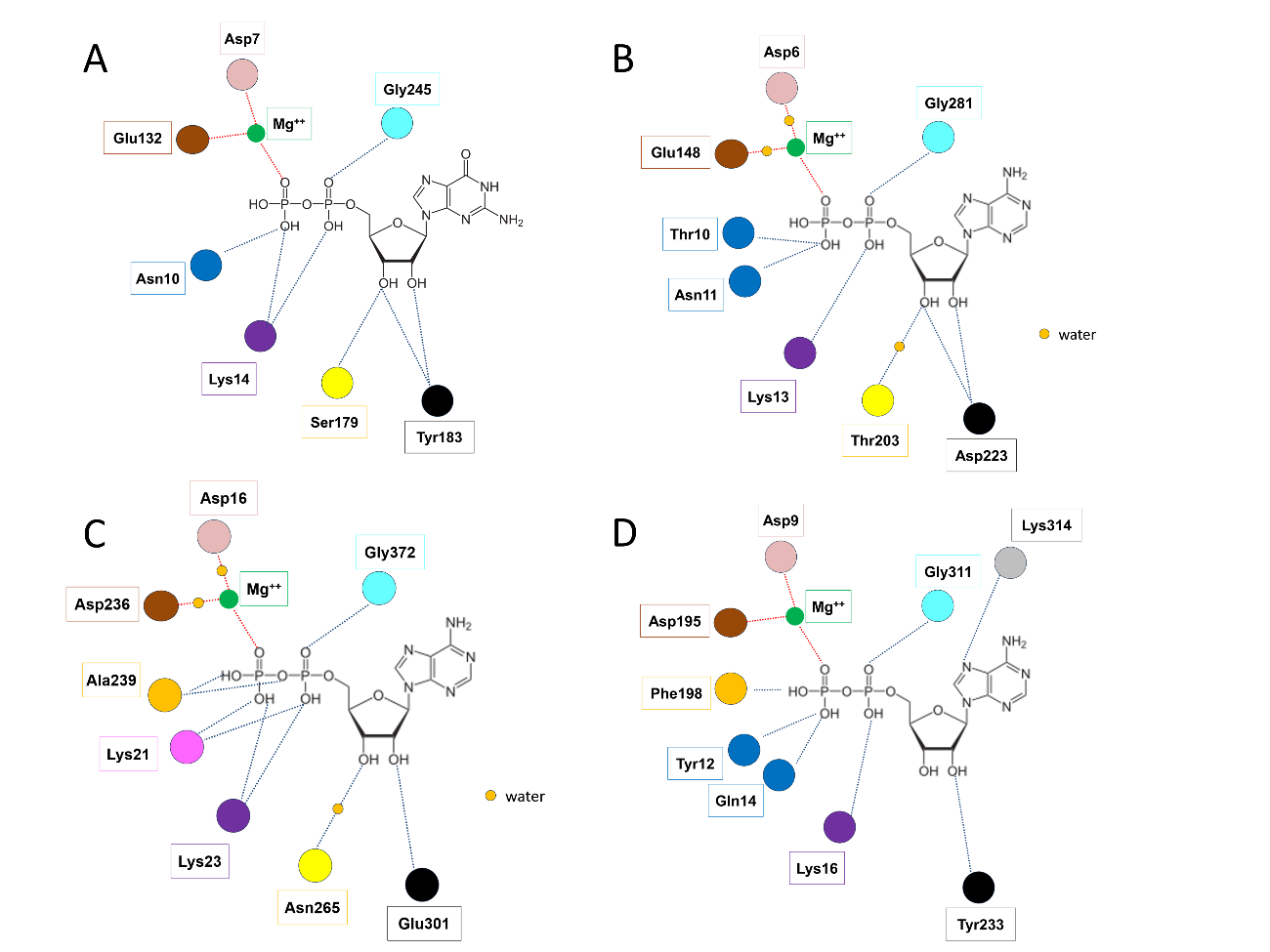
**

**Figure S8: A schematic diagram displaying the nucleotide recognition of the surrounding amino acids for (A)** GDP state of *Cb*-cParM in the filament and **(B)** ParM R1 ADP state crystal structure (*1mwm*). **(C)** BtPartM with ADP (4xe8) (D) CBg-ParM crystal with ADP (6IXW). Comparing with other ParMs, the amino acids surrounding the GDP-bound state of *Cb*-cParM were similar to those of ParM-R1 ADP state crystal structure (*1mwm*).


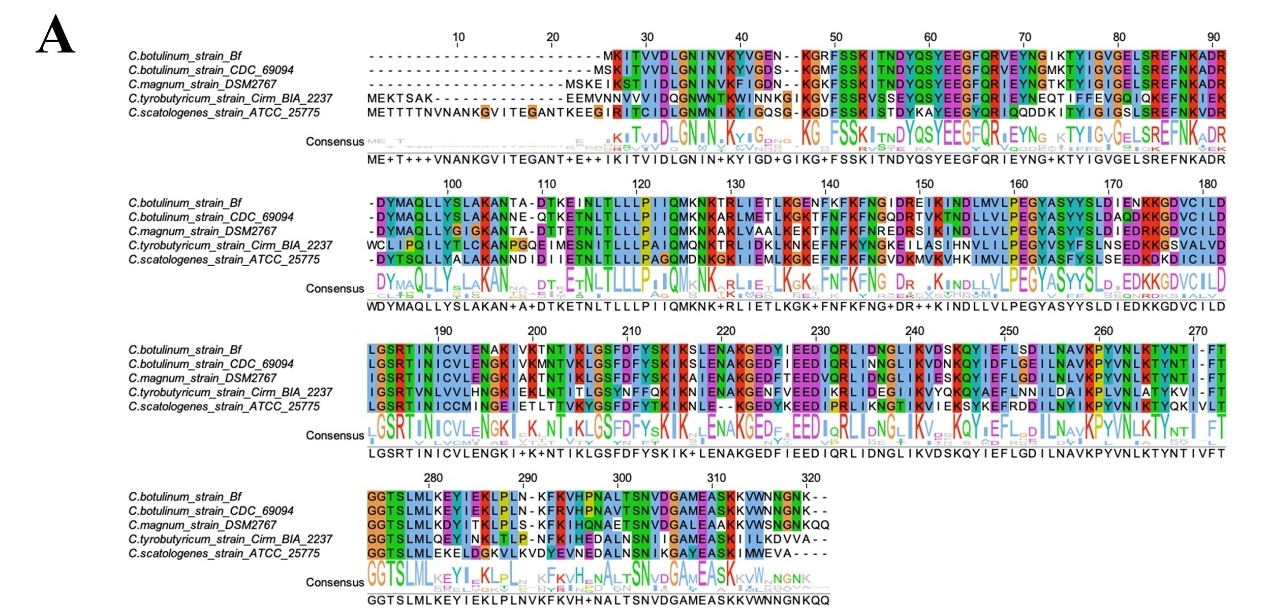


**
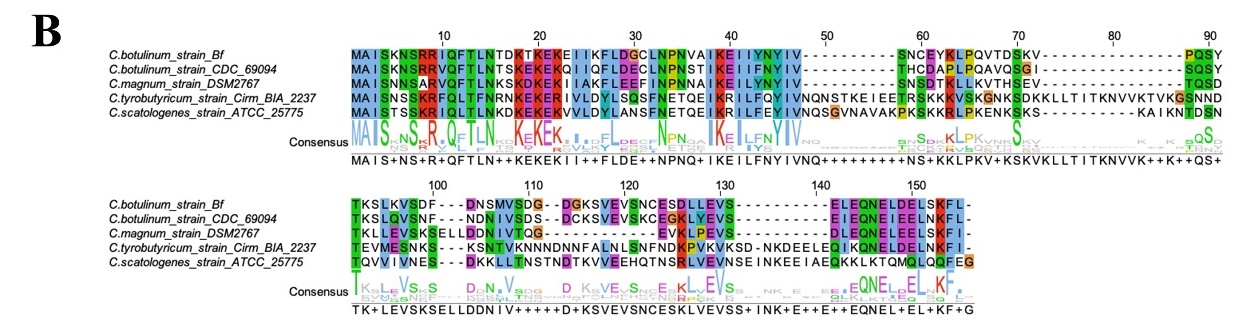
**

**
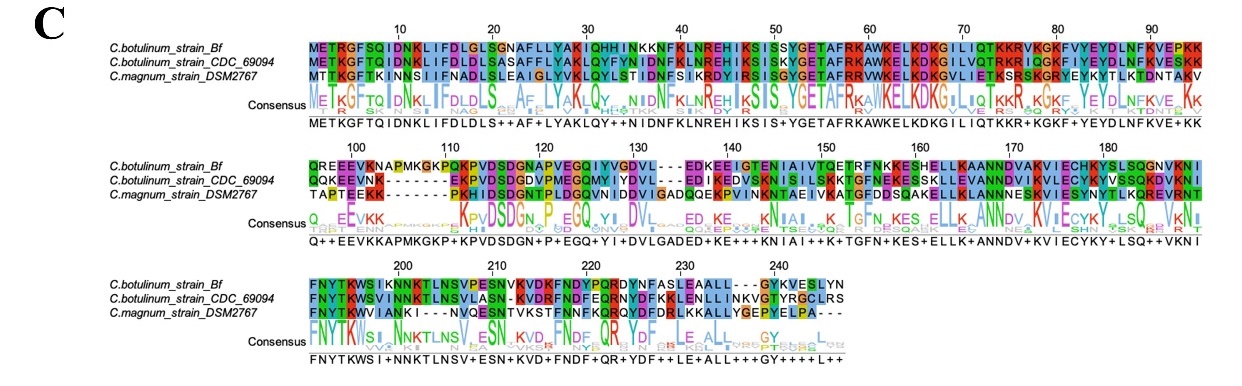
**

**
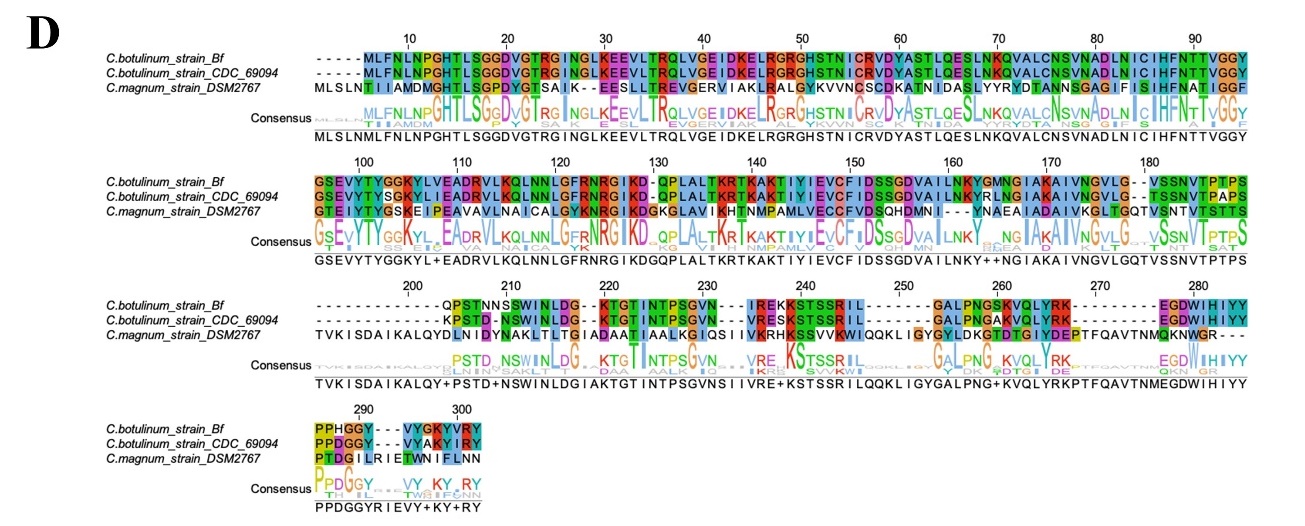
**

**Figure S9: Multiple sequence alignments of homologous sequences of cParCMR gene clusters in *Clostridium sp*.** Multiple sequence alignment of cParM **(A)**, cParR (B), putative replication initiator **(C),** and sporulation-specific N-acetylmuramoyl-L-alanine amidase **(D)** (one of the genes) were performed with MUSCLE (Edgar, 2004). Homologous residues are colored according to the ClustalX scheme.

**
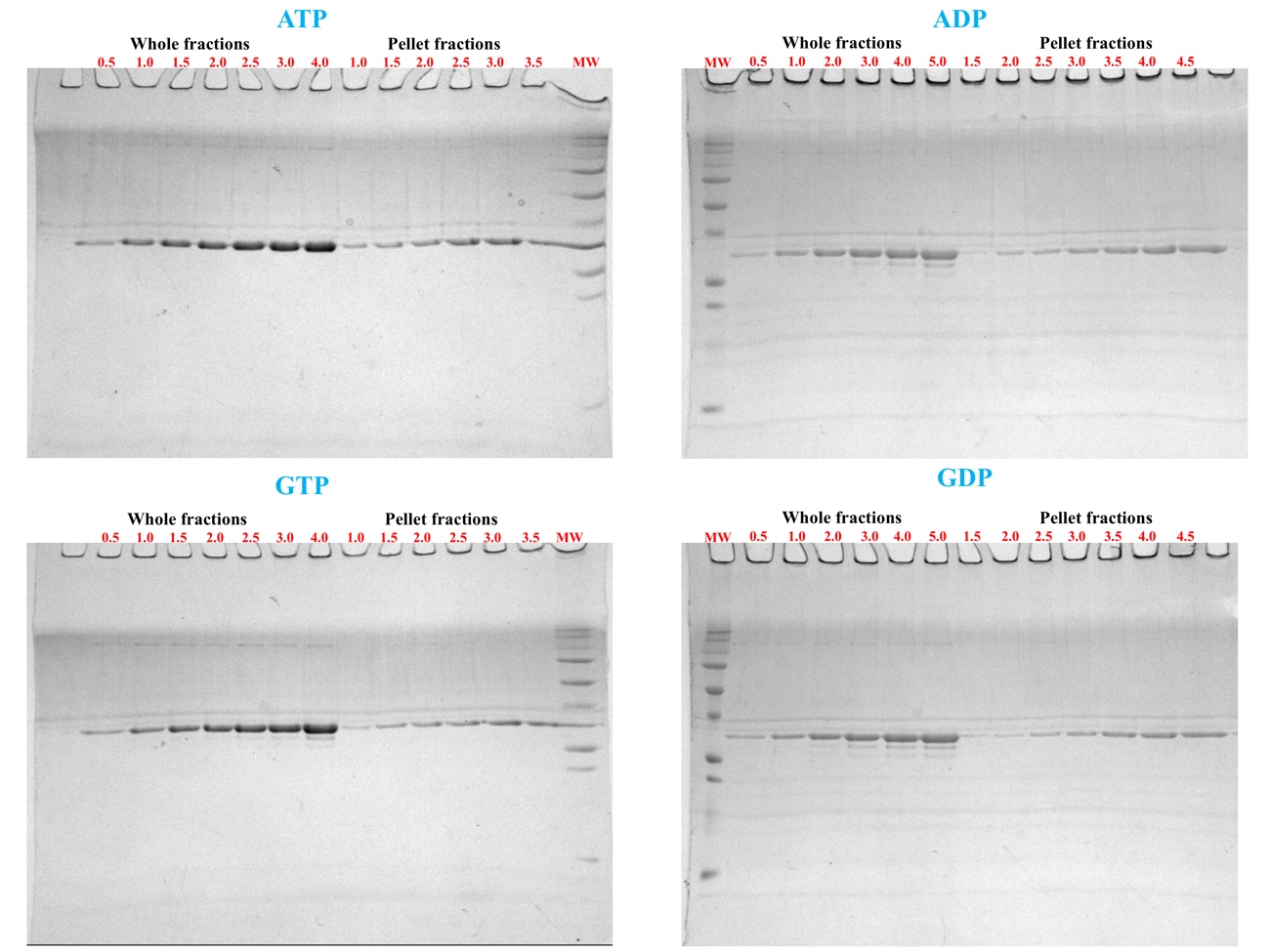
**

**Figure S10: Pelleting assay for determining critical concentrations of *Cb-cParM*.** Varying concentrations of ParM were polymerized with 5 mM nucleotide (ATP, ADP, GTP, or GDP) in 40 mM HEPES (pH 7.5), 150 mM KCl, 2 mM MgCl_2_, and 0.5 mM DTT at 24 °C for 30 min. The standard curve was determined using whole fractions of known concentrations ParM reaction mixture which was used to construct the critical concentration graphs in Fig. S11.


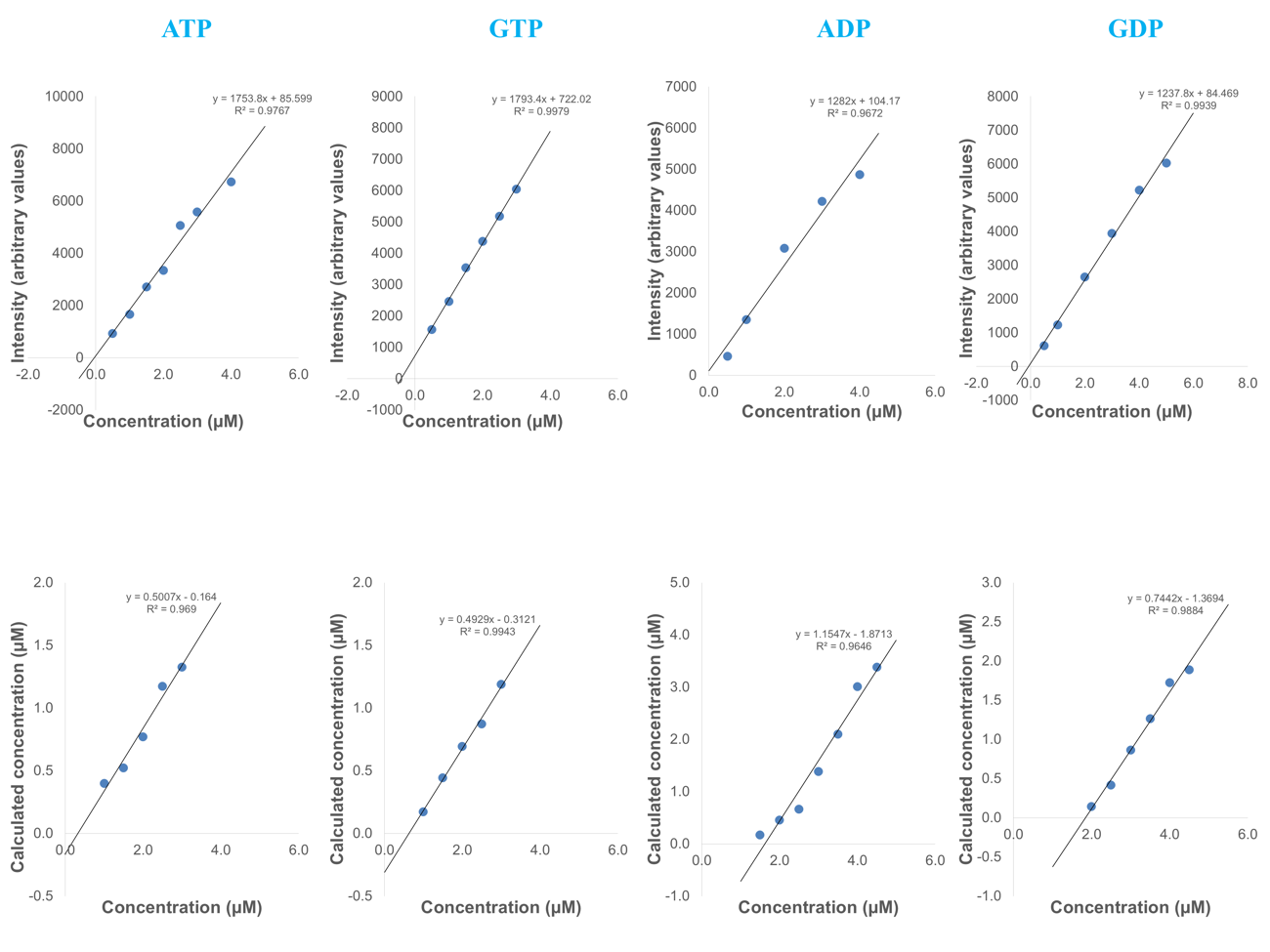


**Figure S11: Determination of critical concentrations of *Cb-cParM***. Whole fractions were used to construct the standard curve (**Upper graphs**) from which the critical concentration graphs (**Lower**) were produced. Varrying concentrations of ParM were polymerized with 5 mM nucleotide (ATP, ADP, GTP, or GDP) in 40 mM HEPES (pH 7.5), 150 mM KCl, 2 mM MgCl_2_, and 0.5 mM DTT at 24 °C for 30 min. The critical concentration marks the concentration at which the graph intersects with the x-axis (concentration).


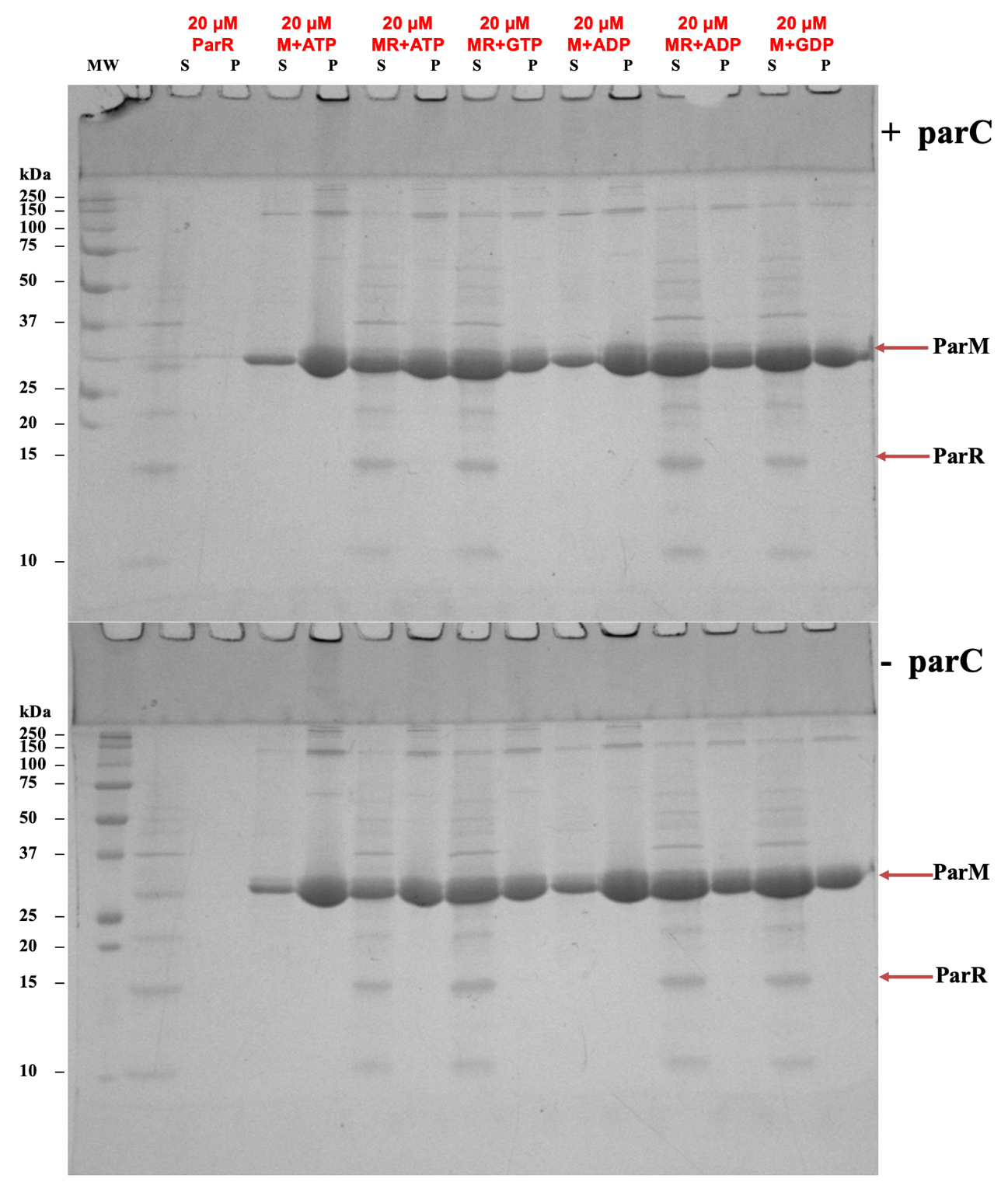


**Figure S12: Sedimentation assay for *Cb-*cParMwith/without *Cb-*cParRand *Cb-cparC* DNA.** M and MR represent *Cb*-cParMwithout or with *Cb*-cParR, respectively. S and P represent supernatant and pellet, respectively. The clear decrease in *Cb*-cParMconcentration in the pellet fraction with *Cb*-cParRindicates the depolymerizing function of *Cb*-cParR. There was no observable difference when *Cb-cparC* DNA was added (**Top gel**) and without *Cb-cparC* DNA (**Lower gel**). This indicates that *cparC*:cParR complex has no effect on the depolymerization property of cParR.

**
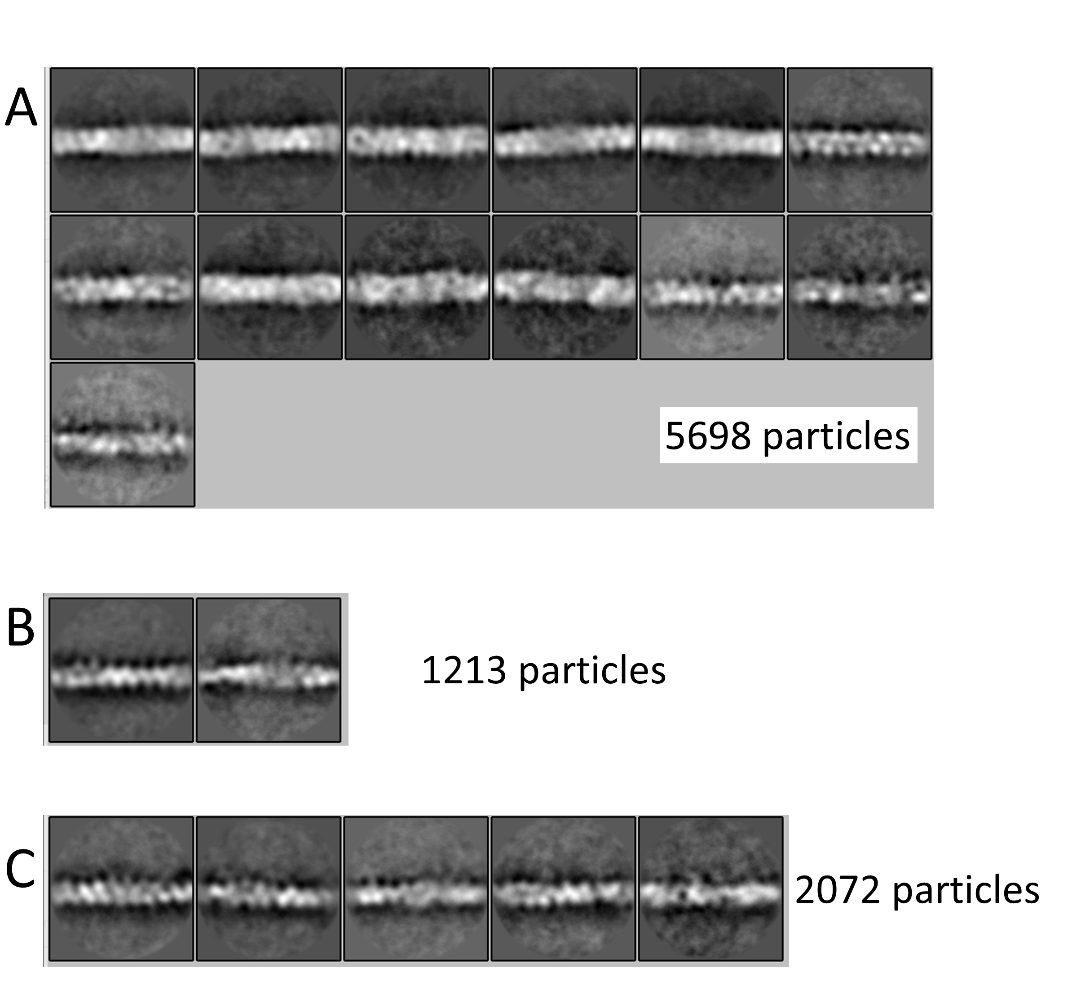
**

**Figure S13: Structural change using nucleotide analogs.** 2D class averages of *Cb-cParM* polymerization using ATP analog, AMPPNP from negatively stained samples. 5 µM *Cb-cParM* was polymerized for 10 minutes with 3 mM of AMPPNP in buffer containing 20 mM HEPES-HCl pH 7.5, 250 mM KCl, 1.7 mM MgCl_2_. The majority of filaments resembled the class 2 type observed with GTP (A, red boxes in **Fig. 6F**) and lacked obvious crossovers. A smaller fraction resembled the class 1 type with GTP (B, blue boxes in **Fig. 6F**). Other filament classes were observed but were difficult to classify (C).

**
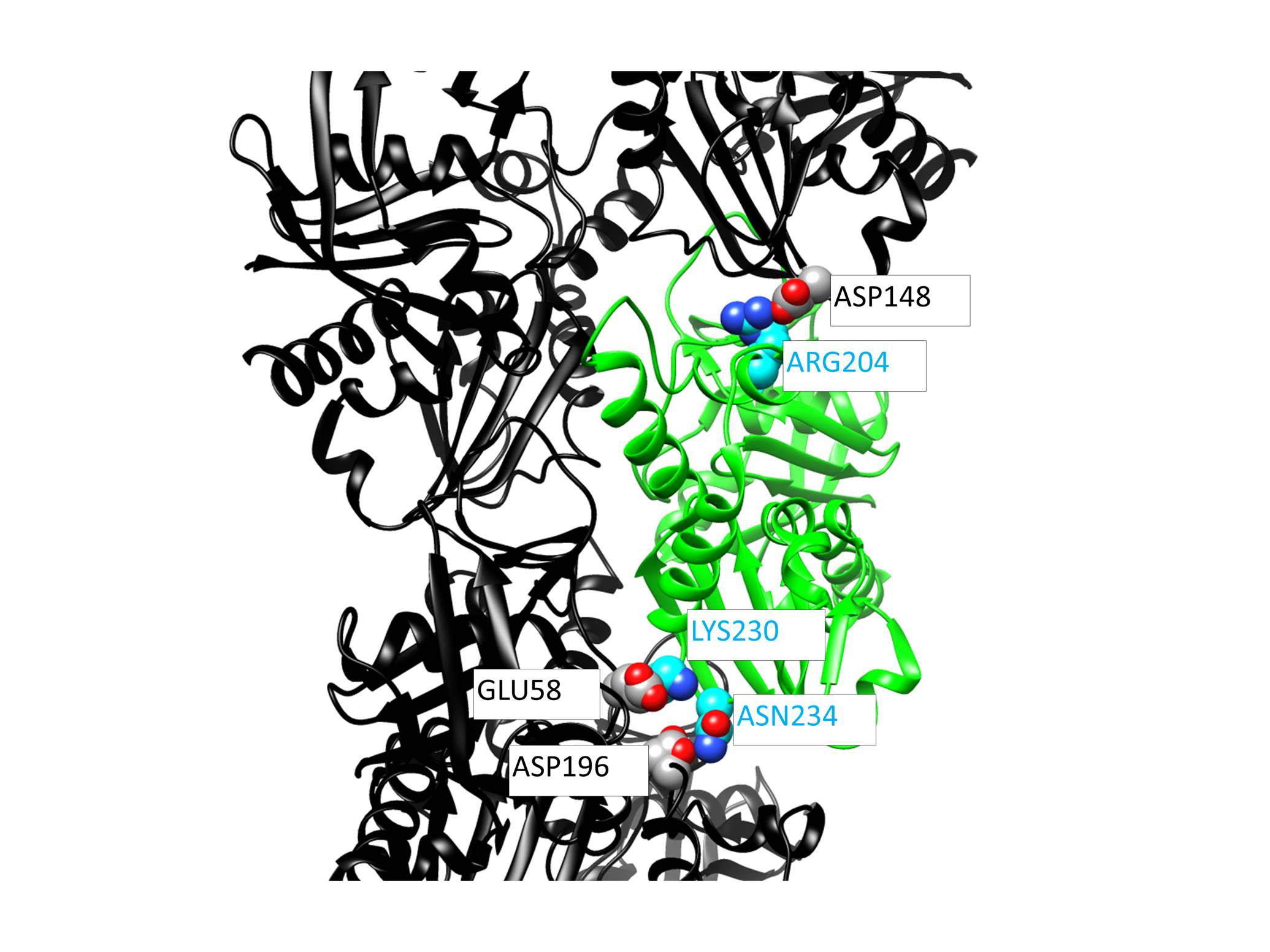
**

**Fig. S14: *Cb*-cParM mutant residues displaying the side chains of R204, K230 and N234.**

**
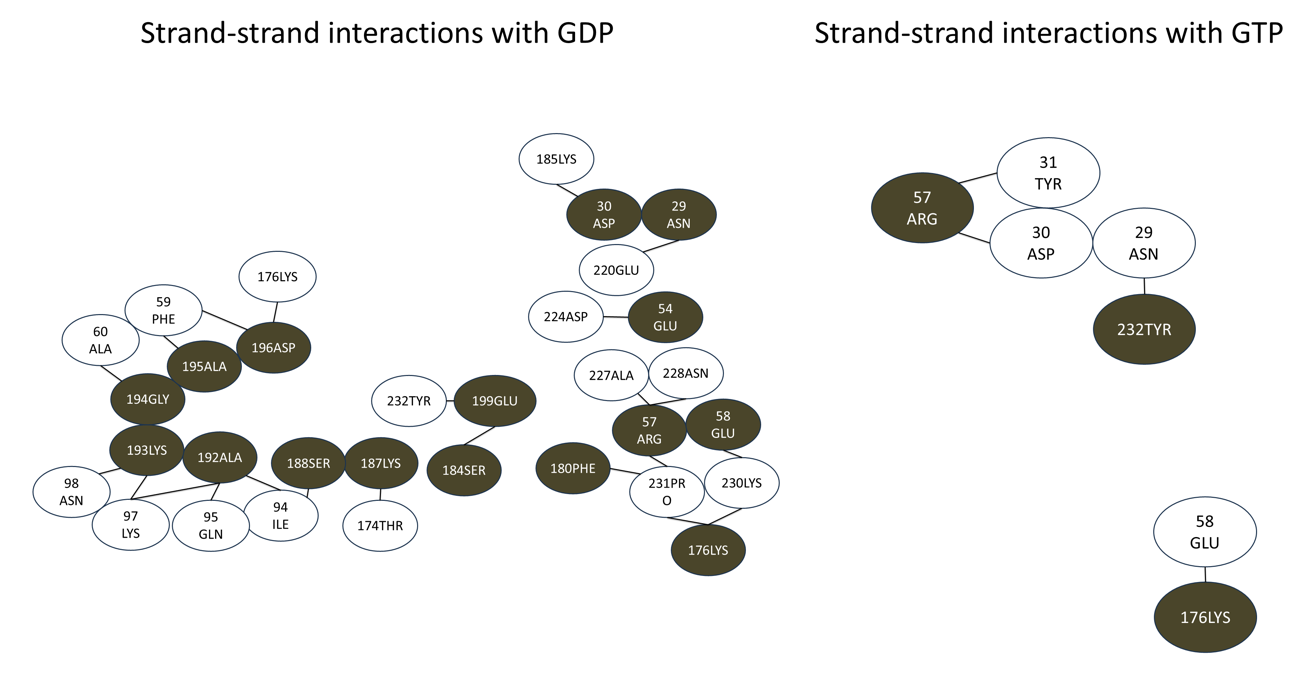
**

**Figure S15: Schematic diagram displaying the strand-strand interactions of *Cb-cParM* with GDP and GTP.** Residues from one strand are colored white and the other are in black.

**Movie S1: Structural shift in *Cb*-cParM filaments.** Models and maps for the class1 (corresponding to GDP state) and the class2 with GTP were aligned by the ID rigid bodies of the center subunit.

**Table S1. Percentage sequence identities of chromosome encoded cParMs**

|  | cParM1  *Natranaerobius*  *thermophilus* | cParM-2  *Natranaerobius thermophilus* | cParM3  *Natranaerobius thermophilus* | *Caloramator* sp | *Clostridium sp* | *Bacillus sp* |
| --- | --- | --- | --- | --- | --- | --- |
| cParM1  *Natranaerobius thermophilus* | 100.0 | 24.9 | <15.0 | 44.6 | 37.8 | 27.5 |
| cParM2  *Natranaerobius thermophilus* | 24.9 | 100.0 | 31.0 | 23.0 | 21.0 | <15.0 |
| cParM3  *Natranaerobius thermophilus* | <15.0 | 31.0 | 100.0 | 23.2 | 26.7 | 27.5 |
| *Caloramator sp* | 44.6 | 23.0 | 23.2 | 100.0 | 46.2 | 30.3 |
| *Clostridium sp* | 37.8 | 21.0 | 26.7 | 46.2 | 100.0 | 27.1 |
| *Bacillus sp* | 27.5 | <15.0 | 27.5 | 30.3 | 27.1 | 100.0 |

**Table S2. Percentage sequence identities of *Moorella* chromosome encoded cParMs**

|  | *Moorella thermoacetica* | *Moorella* sp. | *Moorella glycerini* | *Thermoanaeo*  *bacteraceae* | *Moorella humiferrea* | *Moorella mulderi* |
| --- | --- | --- | --- | --- | --- | --- |
| *Moorella thermoacetica* | 100.0 | 95.7 | 94.8 | 92.5 | 94.2 | 94.2 |
| *Moorella* sp. | 95.7 | 100.0 | 93.7 | 92.0 | 96.8 | 93.1 |
| *Moorella glycerini* | 94.8 | 93.7 | 100.0 | 92.8 | 92.8 | 93.7 |
| *Thermoanaeo*  *bacteraceae* | 92.5 | 92.0 | 92.8 | 100.0 | 90.8 | 91.1 |
| *Moorella humiferrea* | 94.2 | 96.8 | 92.8 | 90.8 | 100.0 | 93.1 |
| *Moorella mulderi* | 94.2 | 93.1 | 93.7 | 91.1 | 93.1 | 100.0 |

**Table S3. Percentage sequence identities of *Bacillus* chromosome encoded cParMs**

|  | cParM1  *Bacillus tropicus* | cParM2  *Bacillus tropicus* | *Bacillus cereus* | *Bacillus anthracis* | *Bacillus paranthracis* | *Bacillus pacificus* |
| --- | --- | --- | --- | --- | --- | --- |
| cParM1  *Bacillus tropicus* | 100.0 | <15.0 | 99.1 | 98.5 | 97.1 | 95.9 |
| cParM2  *Bacillus tropicus* | <15.0 | 100.0 | <15.0 | <15.0 | <15.0 | <15.0 |
| *Bacillus cereus* | 99.1 | <15.0 | 100.0 | 98.8 | 96.5 | 96.5 |
| *Bacillus anthracis* | 98.5 | <15.0 | 98.8 | 100.0 | 96.5 | 96.5 |
| *Bacillus paranthracis* | 97.1 | <15.0 | 96.5 | 96.8 | 100.0 | 99.4 |
| *Bacillus pacificus* | 97.1 | <15.0 | 96.5 | 96.5 | 99.4 | 100.0 |

**Table S4: Statistics of *Dh*-cParM1 protomer, cryoEM and model refinement data**

|  | *Dh*-cParM1 (EMD-37996) (PDB 8X1I) |
| --- | --- |
| **Data collection and processing** |  |
| Voltage (kV) | 300 kV |
| Electron exposure (e–/Å^2^) | 40.0 |
| Defocus range (μm) | ∼ −1.5 −2.3 |
| Pixel size (Å) | 1.1 |
| Phase plate | No |
| Symmetry imposed | Helical |
| Final particle images (no.) | 71060 |
| Map resolution (Å)  FSC threshold | 4.0  0.143 |
| Map resolution range (Å) | ∞ ~ 4.0 |
|  |  |
| **Refinement** |  |
| Initial model used (PDB code) | 8X1I |
| Map sharpening *B* factor (Å^2^) | -241 |
| Model composition  Non-hydrogen atoms  Protein residues  Ligands | 2882  369  2 |
| R.m.s. deviations  Bond lengths (Å)  Bond angles (°) | 0.012  1.973 |
| Validation  MolProbity score  Clashscore  Poor rotamers (%) | 1.21  0.0  3 |
| Ramachandran plot  Favored (%)  Allowed (%)  Disallowed (%) | 95  5  0 |

**Table S5. Cryo-EM data collection, refinement, and validation statistics**

|  | # ADP  (EMDB-33007)  (PDB 7X54) | #2 GDP  (EMDB-33009)  (PDB 7X56) | #3 GTP class 2  (EMDB-33012)  (PDB 7X59) | #4 GTP short incubation  (EMDB-33008)  (PDB 7X55) |
| --- | --- | --- | --- | --- |
| **Data collection and processing** |  |  |  |  |
| Voltage (kV) | 300 kV | 300 kV | 300 kV | 300 kV |
| Electron exposure (e–/Å^2^) | 45 | 45 | 45 | 45 |
| Defocus range (μm) | -1.5 ~ -3.5 | -1.0 ~ -3.0 | -1.0 ~ -3.0 | -0.5 ~ -1.5 |
| Pixel size (Å) | 0.87 | 0.87 | 0.87 | 0.87 |
| Phase plate | No | No | No | Yes |
| Symmetry imposed | Helical | Helical | Helical | Helical |
| Final particle images (no.) | 36762 | 40599 | 70754 | 153326 |
| Map resolution (Å)  FSC threshold | 3.9  0.143 | 3.5  0.143 | 6.5  0.143 | 8.6  0.143 |
| Map resolution range (Å) | ∞ ~ 3.9 | ∞ ~ 3.5 | ∞ ~ 6.5 | ∞ ~ 8.6 |
|  |  |  |  |  |
| **Refinement** |  |  |  |  |
| Initial model used (PDB code) | 7X56 | 6IZV | 7X56 | 7X56 |
| Map sharpening *B* factor (Å^2^) | -105 | -117 | -527 | -1281 |
| Model composition  Non-hydrogen atoms  Protein residues  Ligands | 11575  1425  10 | 11580  1425  10 | 11595  1425  5 | 11600  1425  5 |
| R.m.s. deviations  Bond lengths (Å)  Bond angles (°) | 0.004  0.979 | 0.005  0.965 | 0.004  1.059 | 0.007  1.137 |
| Validation  MolProbity score  Clashscore  Poor rotamers (%) | 1.98  8.79  0.39 | 1.96  7.71  0.00 | 2.19  11.95  0 | 2.44  21.32  0 |
| Ramachandran plot  Favored (%)  Allowed (%)  Disallowed (%) | 91  9  0 | 90  10  0 | 88  12  0 | 89  11  0 |

**Table S6. X-ray crystallography data collection, refinement and validation statistics**

|  | *Cb*-cParM  (PDB code 7X3H) |
| --- | --- |
| **Protein**  Accession No.  Mutations | EDT87363.1  R204D, K230D, N234D |
| **Crystal** | P2_1_ |
| *a*, *b*, *c* (Å) | 55.6, 51.1, 64.9 |
| *   γ* (°) | 90.0, 115.3, 90.0 |
| **Data collection** |  |
| Wavelength (Å) | 1.0 |
| Resolution (Å)^a^ | 50.0-1.7 (1.73-1.70) |
| *R*_merge_ | 3.0 (47.3) |
| *R*_meas_ | 3.5 (58.8) |
| *R*_pim_ | 1.8 (34.4) |
| *I/*σ(*I*) | 37.7 (1.8) |
| *CC*_1/2_ | (0.736) |
| Completeness (%) | 99.3 (94.1) |
| Redundancy | 3.6 (2.5) |
|  |  |
| **Refinement** |  |
| Resolution (Å) | 32.0-1.7 (1.76-1.70) |
| No. reflections | 35734 (2896) |
| *R*_work_ / *R*_free_ | 19.2/22.4 (27.1/29.9) |
| No. atoms  Protein | 2249 |
| Water | 314 |
| *B* factors |  |
| Protein | 25.4 |
| Water | 34.0 |
| r.m.s deviations |  |
| Bond lengths (Å) | 0.007 |
| Bond angles (°) | 1.09 |
| Ramachandran Plot |  |
| Favoured (%) | 98.5 |
| Outliers (%) | 0 |

**References**

1. Edgar RC (2004) MUSCLE: multiple sequence alignment with high accuracy and high throughput. *Nucleic Acids Res* 32: 1792-1797
2. Cameron L M Gilchrist, Yit-Heng Chooi, clinker & clustermap.js: automatic generation of gene cluster comparison figures, Bioinformatics, Volume 37, Issue 16, August 2021, Pages 2473–2475, <https://doi.org/10.1093/bioinformatics/btab007>
3. Edgar, R.C. MUSCLE: a multiple sequence alignment method with reduced time and space complexity. BMC Bioinformatics 5, 113 (2004). <https://doi.org/10.1186/1471-2105-5-113>
4. Waterhouse AM, Procter JB, Martin DMA, Clamp M, Barton GJ (2009) Jalview Version 2 - A multiple sequence alignment editor and analysis workbench. Bioinformatics 25 1189-1191.
5. Steinegger, M., Söding, J. MMseqs2 enables sensitive protein sequence searching for the analysis of massive data sets. Nat Biotechnol 35, 1026–1028 (2017). <https://doi.org/10.1038/nbt.3988>
